# SLAMF7 regulates goblet cell mucus production and negatively impacts gut homeostasis and commensalism

**DOI:** 10.1080/19490976.2025.2527857

**Published:** 2025-07-11

**Authors:** Dianrong Zhou, Changbu Wu, Chengjuan Li, Meisong Li, Zeyu Li, Jiachen Li, Yihao Zhang, Hui Zhao, Yaxuan Wang, Lujie Liang, Lin Xu, Yaxin Li, Lan-Lan Zhong, Siyuan Feng, Guo-Bao Tian

**Affiliations:** a Department of Immunology, School of Medicine, Shenzhen Campus of Sun Yat-sen University, Shenzhen, Guangdong, China; b Advanced Medical Technology Center, The First Affiliated Hospital, Zhongshan School of Medicine, Sun Yat-sen University, Guangzhou, China; c Program in Pathobiology, The Fifth Affiliated Hospital, Zhongshan School of Medicine, Sun Yat-Sen University, Guangdong, China; d Ministry of Education, Key Laboratory of Tropical Diseases Control (Sun Yat-sen University), Guangzhou, China; e School of Basic Medical Sciences, Xizang Minzu University, Xianyang, Shanxi, China; f Division of Molecular Oncology, Graduate School of Medicine, Nagoya University, Nagoya, Aichi, Japan; g School of Pharmacy, Guangzhou Xinhua University, Guangzhou, China

**Keywords:** Inflammatory bowel disease, SLAMF7, microbiota, mucosal barrier, C1Q, macrophage polarization

## Abstract

Crosstalk between the intestinal mucosal barrier and the gut microbiota contributes to maintaining intestinal homeostasis. Accumulating evidence suggests that diverse mechanisms are involved in maintaining intestinal homeostasis. Any disturbance in these pathways can compromise gut homeostasis and trigger chronic inflammatory diseases such as inflammatory bowel disease (IBD). However, how host factors regulate the intestinal mucosal barrier and change the gut microbiome has not been well defined. Here, we discovered that disruption of SLAMF7 protects against intestinal inflammation. SLAMF7 deficiency significantly altered the intestinal microbiota composition, specifically the expansion of the mucus-specific bacterium *Akkermansia muciniphila*. Moreover, SLAMF7 deficiency resulted in goblet cell generation by increasing the number of M2-like C1q+ macrophages, which may contribute to a thicker mucosal barrier. Mechanistically, SLAMF7 deficiency increased goblet cell generation through C1q+ M2-like macrophage polarization, which partly led to a thicker mucosal barrier. Depletion of SLAMF7 in intestinal macrophages upregulated C1q via activation of the STAT6-MafB pathway. The upregulation of C1q in macrophages resulted in a bias toward the M2 phenotype in response to damage-associated molecular patterns (DAMPs) stimulation. Accordingly, SLAMF7 activation induced a shift in macrophage polarization and reduced mucus secretion, which partially aggravated intestinal inflammation. Conversely, SLAMF7 knockdown mitigated DSS-induced intestinal inflammation to some extent. This work reveals the previously unrecognized functions of SLAMF7 in regulating intestinal inflammation and tissue homeostasis.

## Introduction

Accumulating evidence suggests that the intestinal immune system, epithelial structure and microbiota are involved in maintaining intestinal homeostasis. Any disturbance in these pathways can compromise gut homeostasis and trigger chronic inflammatory diseases such as IBD.^1,2^ IBD, including Crohn’s disease and ulcerative colitis, manifests as chronic inflammation of the gastrointestinal tract, profoundly affecting patients’ quality of life. In 2019, there were 40,998 deaths globally from IBD, representing a 68.75% increase from the 24,295 deaths recorded in 1990.^3^ Current treatments for IBD primarily focus on suppressing the immune response to reduce inflammation. Biological therapies, including monoclonal antibodies against tumor necrosis factor-alpha (TNF-α) and integrin receptors, have revolutionized IBD management by inducing remission in a subset of patients. Nonetheless, IBD exhibits relapsing‒remitting patterns with highly heterogeneous phenotypes. Several clinical studies have reported that 20–40% of IBD patients fail to respond to anti-TNF-α induction therapy, whereas nearly 50% of those with an initial response may lose responsiveness to anti-TNF-α maintenance therapy following the induction phase.^4,5^ Current therapeutic limitations stem from an incomplete understanding of the molecular pathogenesis, leading to frequent treatment failure. Personalized medicine requires the precise administration of targeted therapies based on disease-driving molecular mechanisms, which could improve remission rates, prevent treatment-related morbidity, and reduce healthcare costs. Despite implementation challenges, developing validated biomarkers remains crucial for advancing personalized IBD management.^6^

Intestinal macrophages, which are traditionally recognized for their role in orchestrating immune responses, are increasingly acknowledged for their nonimmunological functions, including epithelial health regulation and mucus layer maintenance.^7,8^ In a healthy state, these macrophages promote the homeostasis of the mucus layer by supporting the proliferation and differentiation of goblet cells, which ensures robust mucin production. However, in IBD, dysregulation of these macrophages compromises mucus integrity.^9^ Aberrantly activated macrophages produce proinflammatory cytokines that impair goblet cell function, reduce mucin secretion, and disrupt the mucus layer, thereby increasing the vulnerability of the epithelium to damage and infection.^10^ Additionally, intestinal macrophages may influence the composition of the gut microbiota, which in turn can impact mucin degradation and mucus layer integrity. Several microorganisms, called “mucus-associated microorganisms”, are able to digest mucin glycan via glycan-degrading enzymes,^11,12^ such as the well-known bacteria *Akkermansia muciniphila*, and colonize the intestinal mucus layer. Aberrant macrophage activation and polarization can contribute to the pathogenesis of IBD by sustaining inflammatory responses, highlighting the importance of understanding the mechanism of macrophage regulation. A deeper understanding of the molecular pathways involved in the differentiation and functions of intestinal macrophages could be helpful for designing new targets to promote long-term remission in patients with IBD.

Signaling lymphocytic activation molecule (SLAM) family receptors, comprising several members from SLAMF1 to SLAMF9, are expressed primarily in immune cells.^13^ Ewing’s sarcoma-associated transcript 2 (EAT-2) and SLAM-associated adaptor protein (SAP) are the primary adaptors for SLAMF receptors. Notably, SLAMF molecules serve as both activating and inhibitory receptors, thus playing crucial regulatory roles in the immune system.^14,15^ SLAMF7, a member of the SLAMF family, is expressed predominantly by hematopoietic cells such as NK cells and macrophages. SLAMF7 contains immunoreceptor tyrosine-based switch motifs (ITSMs) in its cytoplasmic domain, which, upon phosphorylation, recruit SH2 domain – containing molecules, including SHP1, SHP2, and SHIP1, to transmit downstream signals.^16^ For example, during polymicrobial sepsis, SLAMF7 negatively regulates sepsis-induced inflammation by diminishing TLR-triggered inflammatory responses through the inhibition of NF-κB and MAPK activation in macrophages.^16^ SLAMF7 has been shown to promote M2 polarization, thereby alleviating corneal inflammation.^17^ Interestingly, a recent study demonstrated that SLAMF7 engagement leads to the superactivation of macrophages under conditions of acute and chronic inflammation, such as rheumatoid arthritis, Crohn’s disease and severe COVID-19 infection.^18^ These studies suggest a link between SLAMF7 and macrophages. Given the crucial role of macrophages in IBD, we hypothesized that SLAMF7 may regulate gut homeostasis.

Here, we showed that SLAMF7 is a negative regulator of intestinal inflammation. SLAMF7 deficiency altered the gut microbiome, specifically promoting mucus-specific bacteria such as *Akkermansia muciniphila*, and protected against DSS-induced intestinal inflammation. Moreover, disruption of SLAMF7 increased goblet cell generation and led to the secretion of excessive mucus by regulating macrophage polarization. Furthermore, SLAMF7 depletion increased C1q levels by activating the STAT6 pathway, which promoted M2 polarization in response to DAMPs stimulation. Consistently, SLAMF7 activation impaired intestinal barrier integrity and increased susceptibility to gut inflammation, whereas SLAMF7 knockdown with siRNA alleviated DSS-induced intestinal inflammation. Taken together, our data highlight the role of SLAMF7 in regulating intestinal macrophage polarization, which is a promising target for remodeling intestinal homeostasis.

## Materials and Methods

### Mice

*Slamf7*^*±*^ mice with a C57BL/6J background were obtained from the Shanghai Research Center of Southern Model Organisms. *Slamf7*^*±*^ mice were raised in our facilities (at the Laboratory Animal Center of Sun Yat-sen University) for two generations, after which *slamf7*^*-/-*^ mice were obtained. WT C57BL/6J and *slamf7*^*-/-*^ mice were raised in the same facility. Intestinal inflammation was induced in 8- to 14-week-old mice. All mice were raised in specific-pathogen-free (SPF) conditions. All animal procedures were approved by the Institutional Animal Care and Use Committee of Sun Yat-sen University.

Detailed methods are provided in the Supplemental Materials and Methods.

## Results

### Loss of SLAMF7 protected against colitis

**Figure 1.**
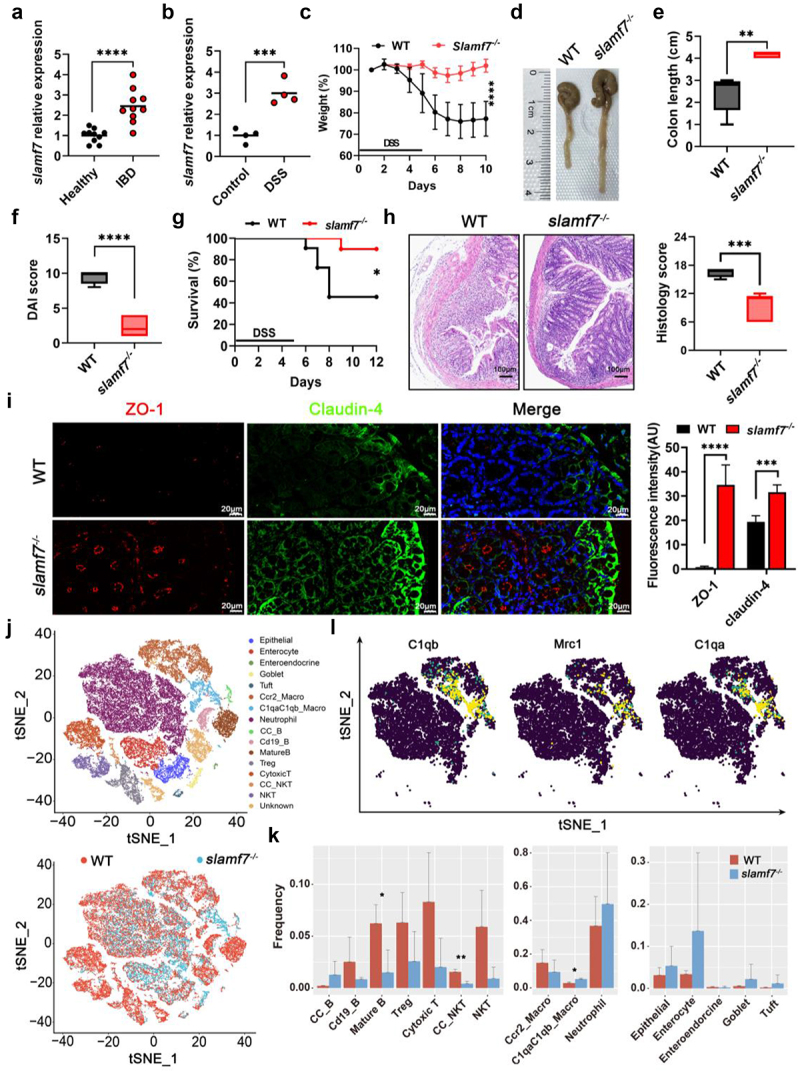
Loss of SLAMF7 offered protection against colitis.

To determine whether SLAMF7 is clinically relevant to intestinal inflammation in humans, we assessed SLAMF7 expression in colon tissue by using publicly available human gene expression data,^19^ which included data from ten healthy donors and ten colitis patients. We found that the expression of SLAMF7 was significantly greater in patients with colitis than in healthy donors (Figure 1(a)). Using DSS-induced colitis in an acute colon tissue inflammation model, we also observed increased SLAMF7 expression during inflammation (Figure 1(b)), suggesting a potential link between SLAMF7 and intestinal inflammation. Because several groups have previously demonstrated that SLAMF7 negatively regulates the inflammatory response in mouse models, we hypothesized that SLAMF7 may contribute to the suppression of intestinal inflammation. Unexpectedly, we observed that wild-type (WT) mice had more severe colitis than did *slamf7*^*-/-*^ mice following DSS treatment. These differences included greater weight loss, shorter colon length, and more signs of clinical disease (measured as the disease-associated index, DAI), as well as increased mortality, in WT mice than in *slamf7*^*-/-*^ mice (Figure 1(c-g)). Additionally, compared with those of WT mice, the colon tissues of *slamf7*^*-/-*^ mice exhibited milder histopathological damages and intact epithelial crypt structures (Figure 1(h)). Given the critical role of tight junction (TJ) proteins in maintaining intestinal epithelial barrier integrity,^20^ we examined the protein expression of ZO-1 and claudin-4. As expected, the expression of TJ proteins was greater in *slamf7*^*-/-*^ mice than in WT mice (Figure 1(i), Figure S1), indicating that SLAMF7 disrupted barrier function in the colon. These results suggest that the absence of SLAMF7 protects against DSS-induced intestinal inflammation.

(a) SLAMF7 mRNA in human colon tissue from healthy donors and IBD patients was assessed; the results are presented relative to those of healthy controls. (b) The relative expression of SLAMF7 in colon tissue from WT mice fed 2% DSS (*n* = 4/group) or sterile water (control, *n* = 4/group) was determined. Intestinal inflammation in DSS-treated *slamf7*^*-/-*^ mice (*n* = 5/group) and WT mice (*n* = 5/group) was monitored on the basis of daily weight loss (c). Representative images of the colon (d). Colon length (e). DAI score (f). Survival (g). (h) H&E staining of histological sections of mouse colons (*n* = 5/group) was performed. The histological score was used to assess the severity of tissue damage and inflammation in the colon sections. Scale bars, 20 μm. (i) Fluorescence staining of mouse colon sections for ZO-1 (red) and claudin-4 (green) was performed. The fluorescence intensities of ZO-1 and claudin-4 (right) were determined. Scale bars, 100 μm. (j) The t-SNE plot displays the 51,976 cells from mouse colons (*n* = 3/group) separated into 15 subsets (upper); t-SNE plot cells are colored according to sample origin (lower). The blue dots indicate the *slamf7*^*-/-*^ group, and the red dots represent the WT group. (k) The bar graphs show the average frequencies of the indicated cell subtypes: T cells, NK cells, plasma cells, myeloid cells and epithelial cells. (l) The t-SNE plot shows the gene expression of C1qaC1qb macrophage populations. The data are presented as the means ± SEMs. *p < 0.05; **p < 0.01; ***p < 0.001; ****p < 0.0001.

To investigate transcriptional signatures that might be specific to *slamf7*^*-/-*^ mice, we performed scRNA-seq to analyze colon tissues from WT (*n* = 3) and *slamf7*^*-/-*^ (*n* = 3) mice during inflammation. Cells from colon tissues were processed into single-cell suspensions, FACS-purified on the basis of the viability dye 7-aminoactinomycin D (7-ADD),^21^ and subjected to scRNA-seq using the 10X Genomics Chromium platform. After quality control, we obtained transcriptomic data for 51,976 cells. According to the marker gene expression data (Figure S2a,b), these cells were partitioned into 15 groups using unsupervised graph clustering (Figure 1(j)) and visualized by *t*-distributed stochastic neighbor embedding (t-SNE). We subclustered lymphocyte cells into 7 subsets according to previously defined marker genes (Figure S2a,b). Dramatic decreases in the populations of T, B and NK cells were observed in the *slamf7*^*-/-*^ mice compared with the WT mice (Figure 1(k), Figure S3a,b). Gene set enrichment analysis (GSEA) revealed that deletion of SLAMF7 was correlated with inhibition of the T-cell pathway (Figure S3c). Furthermore, flow cytometry confirmed the decrease in lymphocyte counts in the *slamf7*^*-/-*^ mice (Figure S3d). These results indicate that the decrease in lymphocyte infiltration might be associated with the absence of SLAMF7. In addition, CC_B cells (joining chain-expressing B cells), which promote mucosal immunity by enhancing the transport of IgA across the intestinal epithelium to the intestinal mucosa,^22^ were more abundant in *slamf7*^*-/-*^ mice than in WT mice (Figure S3e,f). We subsequently subclustered myeloid cells into 3 subsets (Figure S4a). Interestingly, C1qa- and C1qb-positive macrophages (C1qaC1qb_Macro) with increased *MRC1* (CD206) expression were significantly enriched in the colon of *slamf7*^*-/-*^ mice (Figure 1(l) and Figure S4b). This subset exhibited increased expression of the anti-inflammatory cytokines *IL-10*, *VEGF-α*, and *TGF-β* (Figure S4c,d), which are critical for mucosal repair.^23^ Next, we found that epithelial cells were more abundant in *slamf7*^*-/-*^ mice than in WT mice (Figure 1(k), Figure S5a). These epithelial cells were subgrouped into five subsets according to the expression of marker genes (Figure S2a). GSEA also revealed that the IGF-1 receptor and longevity pathway, which is involved in epithelial repair,^24^ was upregulated in the *slamf7*^*-/-*^ mice compared with the WT mice (Figure S5b). The DEGs in goblet cells from WT and *slamf7*^*-/-*^ mice were involved mainly in the synthesis and transport of glycoproteins (Figure S5c). These results suggest a potential association between SLAMF7 and delayed mucosal repair under inflammatory intestinal conditions.

### slamf7^−/−^ mice were protected from colitis in a microbiota-dependent manner

**Figure 2.**
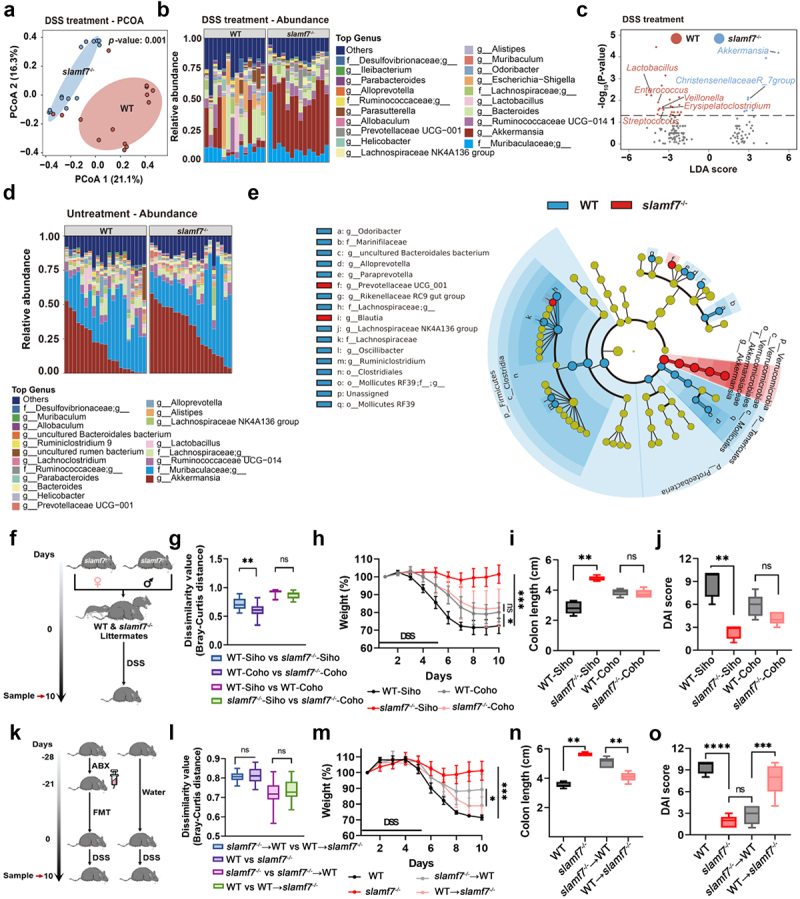
The colons of the *slamf7*^*-/-*^ mice were enriched with *Akkermansia muciniphila* and presented less epithelial damage.

Previous studies have suggested that SLAMF receptors play a role in responses to the gut microbiota.^25,26^ Given the important role of the gut microbiota in intestinal inflammation,^27^ we speculated that SLAMF7 may alter the microbiome during intestinal inflammation. Next, we assessed how SLAMF7 affects susceptibility to colonic inflammation. 16S rRNA sequencing of fecal samples from DSS-treated mice revealed that, compared with those from WT mice, the gut microbiota of *slamf7*^*-/-*^ mice was distinct (Figure 2(a)). Relative abundance analysis revealed that bacteria from the genera *Akkermansia* and *Christensenellaceae R_7* were enriched in *slamf7*^*-/-*^ mice, whereas *Enterococcus*, *Streptococcus*, *Erysipelatoclostridium*, *Veillonella*, and *Lactobacillus* were enriched in WT mice (Figure 2(b,c)). Previous studies have reported *Akkermansia muciniphila* and *Christensenellaceae R_7* as signatures of a healthy gut,^28,29^ and *Enterococcus*, *Streptococcus*, *Erysipelatoclostridium*, and *Veillonella* have been linked to gut disorders.^30^ We observed that patients with IBD exhibit a microbiome profile distinct from that of DSS-treated *slamf7*^*-/-*^ mice (Figure S6a). These findings suggest the presence of a protective microbiome in *slamf7*^*-/-*^ mice during intestinal inflammation. This finding raises the possibility that SLAMF7 deficiency alters the microbiome at steady state (untreated with DSS). To test this hypothesis, we collected feces from WT and *slamf7*^*-/-*^ mice at steady state and analyzed the bacterial communities. The community composition of the *slamf7*^*-/-*^ mice was different from that of the WT mice. Changes in environmental conditions, such as housing and diet, are key factors that can influence the gut microbiota.^31^ Thus, microbiome analysis of mice housed in a second vivarium was performed with four independent biological replicates, and similar results were obtained (Figure S6b). Relative abundance analysis revealed that the abundance of bacteria from the genus *Akkermansia* was increased in *slamf7*^*-/-*^ mice (Figure 2(d)). Linear discriminant analysis effect size (LEfSe) determination further confirmed that *Akkermansia muciniphila* was enriched in *slamf7*^*-/-*^ mice (Figure 2(e)). These results suggest that disruption of SLAMF7 is correlated with *Akkermansia muciniphila* expansion. Moreover, untargeted metabolomic analysis revealed that the abundance of indole derivatives, such as indoleacetic acid, indole-3-carboxylic acid, kynurenic acid, and indoleacetaldehyde, was increased in the feces of *slamf7*^*-/-*^ mice (Figure S6c,d). These results further demonstrate that the microbiome of *slamf7*^*-/-*^ mice was altered at steady state.

(a) Principal coordinate analysis (PCoA) of the fecal microbiota was performed on the basis of the Bray‒Curtis distance and the relative abundance of bacterial genera (b) in WT (*n* = 14) and *slamf7*^*-/-*^ mice (*n* = 14) after DSS treatment. (c) Volcano plot showing the differentially abundant genera in the fecal microbiota of WT and *slamf7*^*-/-*^ mice after DSS treatment. The genera that were enriched in WT and *slamf7*^*-/-*^ mice are marked by red and blue dots, respectively. (d) The relative abundance of bacterial genera in WT (*n* = 21) and *slamf7*^*-/-*^ mice (*n* = 21) at steady state were determined. (e) Cladogram showing the results of LEfSe analysis of differentially abundant bacteria in WT and *slamf7*^*-/-*^ mice. (f) Scheme depicting the littermate cohousing experimental design. Littermate mice originating from the same breeders were divided into groups that were either housed separately (WT-Siho and *slamf7*^*-/-*^-Siho as controls: *n* = 5/group) or cohoused (WT-Coho and *slamf7*^*-/-*^-Coho, *n* = 6/group). (g) Microbiome dissimilarity values were determined on the basis of the Bray‒Curtis distances of separately bred and cohoused littermate WT and *slamf7*^*-/-*^ mice. (h-j) The body weight (h), colon length (i), and DAI score (j) of the mice in (f) were assessed after DSS treatment. (k) The scheme depicts the FMT experiment. *slamf7*^*-/-*^→WT mice (*n* = 5) were orally gavaged with a suspension of feces derived from *slamf7*^*-/-*^ mouse donors (*n* = 5), and WT→*slamf7*^*-/-*^ mice (*n* = 5) were orally gavaged with a suspension of feces derived from WT mouse donors (*n* = 5). WT and *slamf7*^*-/-*^ mouse donors were used as controls. (l) Microbiome dissimilarity values were determined on the basis of the Bray‒Curtis distances of WT and *slamf7*^*-/-*^ mice and between WT mice and *slamf7*^*-/-*^ mice following FMT. (m-o) The body weight (m), colon length (n), and DAI score (o) of the mice in (k) were assessed after DSS treatment. The data are presented as the means ± SEMs. ns, not significant; *p < 0.05; **p < 0.01; ***p < 0.001; ****p < 0.0001.

We next investigated whether the distinct microbiome of *slamf7*^*-/-*^ mice was involved in susceptibility to colitis by feeding mice a broad-spectrum antibiotic cocktail (ABX) (Figure S7a,b). After ABX treatment, no differences in weight loss, colon length, or clinical disease score were observed between WT and *slamf7*^*-/-*^ mice (Figure S7c-e), indicating a relationship between the intestinal microbiota and SLAMF7-mediated exacerbation of colitis. To determine whether the altered microbiota in *slamf7*^*-/-*^ mice was essential for protecting these mice from colitis, we bred heterozygous *slamf7*^*±*^ mice together so that the WT and *slamf7*^*-/-*^ offspring were exposed to the same microbiota throughout development and adulthood (Figure 2(f)). There were differences in the microbiomes of the separately bred WT-Siho and *slamf7*^*-/-*^-Siho mice. In contrast, there was no difference in the microbiomes of WT-Coho and *slamf7*^*-/-*^-Coho (Figure 2(g)). Importantly, we found that, compared with separately bred WT-Siho mice, separately bred *slamf7*^*-/-*^-Siho mice presented a greater abundance of *Akkermansia muciniphila*, while the abundance of this bacterium was comparable between WT-Coho and *slamf7*^*-/-*^-Coho mice (Figure S7f). We then challenged these cohoused mice and separately bred WT-Siho and *slamf7*^*-/-*^-Siho mice with DSS. WT-Coho and *slamf7*^*-/-*^-Coho mice presented comparable results in terms of weight loss, colon length, and clinical disease scores (Figure 2(h-j)). However, separately bred WT-Siho mice displayed more severe colitis than WT-Coho mice, indicating that, to some extent, WT-Coho mice were protected from acute colitis compared with separately bred WT-Siho mice.

To further validate whether the altered microbiome observed in *slamf7*^*-/-*^ mice may be responsible for the reduction in colon inflammation, we colonized ABX-treated WT and *slamf7*^*-/-*^ mice with microbiota from either WT or *slamf7*^*-/-*^ mice and analyzed the effects of fecal microbiota transplantation (FMT) (Figure 2(k)). Consistent with the results above, we observed that the microbiota composition of WT recipients after FMT (*slamf7*^*-/-*^→WT) was more similar to the microbiota composition of *slamf7*^*-/-*^ mice and was distinct from that of WT mice and *slamf7*^*-/-*^ recipients after FMT (WT→*slamf7*^*-/-*^) (Figure 2l). Additionally, a high abundance of *Akkermansia muciniphila* was observed in *slamf7*^*-/-*^→WT mice, but its abundance was lower than that in *slamf7*^*-/-*^ mice, indicating that *slamf7*^*-/-*^ mice may be selectively colonized with *Akkermansia muciniphila* (Figure S7g). We then challenged these recipient mice with DSS and found that the microbiota of the *slamf7*^*-/-*^ mice partially prevented weight loss, colon shortening, and histological damage (Figure 2(m-o)), suggesting that the microbiota of *slamf7*^*-/-*^ mice was required for the reduced susceptibility of WT mice to DSS-induced colitis.

To examine whether *Akkermansia muciniphila* was specifically enriched in *slamf7*^*-/-*^ mice, we established an intestinal microbiome rebuilding model in mice. After ABX treatment, we detected no difference in the intestinal microbiome composition between WT and *slamf7*^*-/-*^ mice after antibiotic exposure (Figure S7h,i). However, the abundance of *Akkermansia muciniphila* was greater in *slamf7*^*-/-*^ mice than in WT mice (Figure S7j). These results suggest that the loss of SLAMF7 is associated with *Akkermansia muciniphila* expansion in the mouse colon.

Taken together, these data suggest that the microbiota of *slamf7*^*-/-*^ mice provides partial protection against colitis.

### Absence of SLAMF7 leads to the production of a thicker colon mucus layer

**Figure 3.**
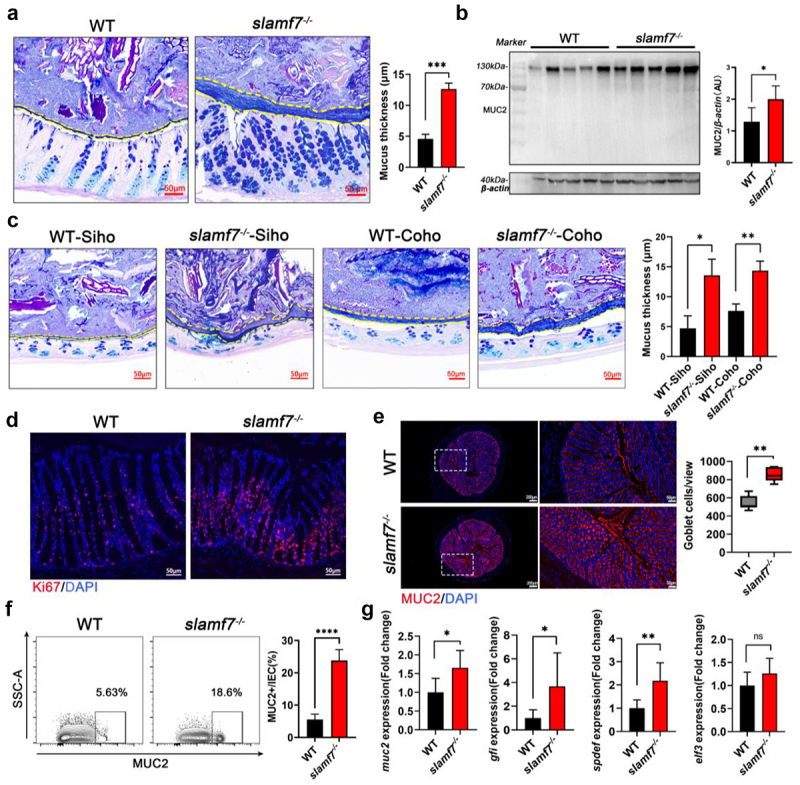
SLAMF7 deficiency led to goblet cell proliferation and excessive mucus production.

*Akkermansia muciniphila* is a mucus-utilizing bacterium that enables mucus digestion by glycan-degrading enzymes.^32^ This finding raised the possibility that the high relative abundance of *Akkermansia muciniphila* in the colon of the *slamf7*^*-/-*^ mice points to the overabundance of mucus in these mice. To test this hypothesis, we examined colon tissues using Alcian blue/periodic acid-Schiff (AB/PAS) staining, which stains glycosylated proteins in goblet cell mucins. The mucus layer was assessed in age- and sex-matched WT and *slamf7*^*-/-*^ mice under steady-state conditions. We found that the colon mucus layer was thicker in the *slamf7*^*-/-*^ mice than in the WT mice (Figure 3(a)). Accordingly, the protein level of the most abundant protein in the intestinal mucus, MUC2, was significantly increased in the colon of the *slamf7*^*-/-*^ mice (Figure 3(b)). To investigate whether the microbiota contributes to the overabundance of mucus in *slamf7*^*-/-*^ mice, we assessed the colon mucous layer thickness in WT-Coho and *slamf7*^*-/-*^-Coho mice (which share similar microbiota). Interestingly, the mucus layer in *slamf7*^*-/-*^-Coho mice was still thicker than that in their WT-Coho littermates (Figure 3c), indicating that the microbiota was not responsible for promoting excessive mucus in *slamf7*^*-/-*^ mice.

(a) AB-PAS staining showing the thickness of the mucus layer in the colon of WT and *slamf7*^*-/-*^ mice at steady state; scale bar, 50 μm. The thickness of the mucus layer was measured via a histogram (right, *n* = 3/group). (b) Western blot analysis was performed to quantify MUC2 expression in the colon of WT and *slamf7*^*-/-*^ mice at steady state, and the quantified results are shown in the histogram (right, *n* = 5/group). (c) AB‒PAS staining showing the thickness of the mucus layer in separately housed (Siho) and cohoused (Coho) littermate mice at steady state; scale bar, 50 μm. The thickness of the mucus layer was measured, and the results are presented in the histogram (right, *n* = 3/group). (d) Ki67 and DAPI were used to stain the colon from WT and *slamf7*^*-/-*^ mice; scale bar, 50 μm. (e) Immunofluorescence staining showing the expansion of goblet cells in the colon from WT and *slamf7*^*-/-*^ mice at steady state. A microscopy image is shown on the left, scale bars = 200 μm. The magnification of the panels is provided on the right; scale bars, 50 μm. MUC2 fluorescence density was quantified (*n* = 5/group). (f) Flow cytometric analysis of goblet cells from the colon of WT and *slamf7*^*-/-*^ mice was performed (*n* = 5/group). (g) qRT‒PCR analysis of the indicated genes in the colon of WT and *slamf7*^*-/-*^ mice was performed. The data are presented as the means ± SEMs. ns, not significant; *p < 0.05; **p < 0.01; ***p < 0.001.

Notably, *slamf7*^*-/-*^ mice presented an increased number of goblet cells per crypt (Figure 3(a)). Furthermore, high expression of the cell proliferation marker Ki67 in epithelial cells was observed in *slamf7*^*-/-*^ mice (Figure 3(d)). This finding led us to hypothesize that excessive mucus production may be a result of goblet cell expansion. To test this hypothesis, colon tissues from WT and *slamf7*^*-/-*^ mice at steady state were collected and stained with antibodies against the goblet cell-specific marker anti-MUC2. We found that the number of goblet cells was significantly greater in *slamf7*^*-/-*^ mice than in WT mice (Figure 3(e)), and these results were confirmed by flow cytometry^33,34^ (Figure 3f). These results suggest that goblet cells from *slamf7*^*-/-*^ mice may undergo active cell proliferation. Indeed, according to the qRT‒PCR results, the transcript levels of *muc2* and transcription factors that mark the goblet cell lineage, *Spdef* and *Gfi1*, were significantly greater in *slamf7*^*-/-*^ mice than in WT mice (Figure 3(g)). These results suggest that SLAMF7 deficiency partially promotes goblet cell expansion and a thicker colon mucus layer.

### Disruption of SLAMF7 promotes colonic macrophage polarization to induce goblet cell proliferation and increase mucus production

**Figure 4.**
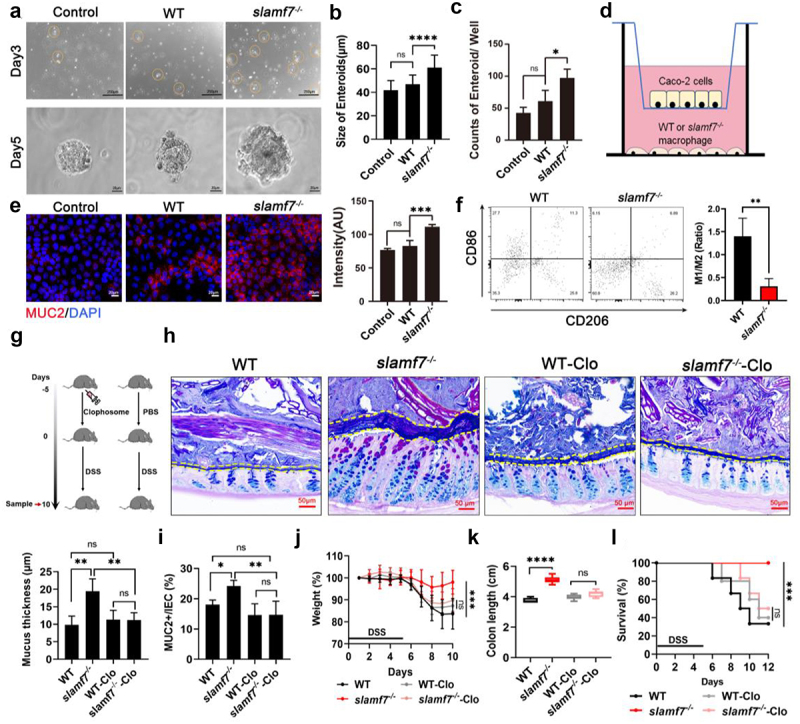
SLAMF7 regulated macrophage polarization to control intestinal inflammation.

What is the mechanism by which SLAMF7 deficiency promotes goblet cell expansion? The SLAMF7 receptor is expressed predominantly by immune cells.^35^ Consequently, goblet cell expansion may not be directly controlled by SLAMF7. CD206+ intestinal macrophages promote mesenchymal niche cell proliferation.^36^ During intestinal inflammation, we observed a significant increase in the C1qaC1qb macrophage population in the colon tissue of *slamf7*^*-/-*^ mice, and this subset displayed high expression of CD206 (Figure 1(k-l)), which led us to hypothesize that the intestinal macrophages from the *slamf7*^*-/-*^ mice play a role in regulating mesenchymal niche cell proliferation at steady state. To test this hypothesis, we isolated intestinal macrophages from WT and *slamf7*^*-/-*^ mice at steady state using fluorescence-activated cell sorting and performed intestinal organoid coculture experiments (Figure 4(a)). Coculture of enteroids with macrophages from *slamf7*^*-/-*^ mice caused the centroid area to expand to 50% more than that of untreated controls (Figure 4(b)) and significantly increased the number of enteroids (Figure 4(c)). In contrast, compared with that of the untreated control, the size of the enteroids cocultured with macrophages from the WT mice was only slightly greater (Figure 4(c)). Furthermore, we collected all of the particles in each well and determined their size by flow cytometry^37^ (Figure S8a,b). These data demonstrate the ability of macrophages from *slamf7*^*-/-*^ mice to promote intestinal stem cell (ISC) proliferation. To further investigate how these macrophages from *slamf7*^*-/-*^ mice control the exact amount of mucus, we used a Transwell insert coculture system based on Caco-2 epithelial cells and intestinal macrophages^38^ isolated from WT and *slamf7*^*-/-*^ mouse intestines (Figure 4(d)). Compared with macrophages from WT mouse intestines, macrophages from *slamf7*^*-/-*^ mouse intestines induced Caco-2 cells to secrete significantly more mucin (Figure 4(e)). These findings suggest that macrophages from *slamf7*^*-/-*^ mice promote the production of excessive mucus by promoting goblet cell growth *in vitro*.

(a) 3D coculture of ISCs with intestinal macrophages from WT and *slamf7*^*-/-*^ mice. The results following coculture for 3 days (upper) are shown; scale bar, 250 μm. The results following coculture for 5 days (below) are shown; scale bar, 20 μm. (b-c) Enteroids were microscopically measured and quantified on day 7. (d) A monolayer of Caco2 cells was cocultured with intestinal macrophages from WT and *slamf7*^*-/-*^ mice. (e) Representative confocal microscopy images of Caco2 cells stained with an anti-MUC2 antibody are shown; scale bar, 20 μm. The fluorescence intensity was quantified (right). (f) Flow cytometric analysis of CD206+ M2 macrophages and CD86+ M1 macrophages in the colon of WT (*n* = 4) and *slamf7*^*-/-*^ (*n* = 4) mice was performed, as shown by representative flow charts (left). The ratio of CD86+ M1 macrophages to CD206+ M2 macrophages was quantified (right). (g) The murine colonic macrophage clearance model is shown. (h) The mucus layer thickness was measured in mice treated with clodronate liposomes without subsequent DSS administration (*n* = 4/group). (i) Flow cytometry analysis of MUC2+ cells in the mouse colon (*n* = 5/group) was performed. (j) The body weight, (k) colon length and survival (l) of the mice described in (g) were determined. The data are presented as the means ± SEMs. *p < 0.05; **p < 0.01; ***p < 0.001; ****p < 0.0001.

In response to changes in their environment, macrophages can become polarized into two functionally different types: M1-like macrophages, which have proinflammatory effects, and M2 macrophages, such as CD206+ macrophages, which have anti-inflammatory, growth-promoting, and healing effects.^7,39^ Given these studies and our observations of the impact of macrophages from *slamf7*^*-/-*^ mice on intestinal stem cells and mucus production, we next explored whether SLAMF7 deficiency promotes macrophage polarization toward an anti-inflammatory phenotype. Indeed, we detected a reduction in the M1/M2 ratio in the colon tissues of the *slamf7*^*-/-*^ mice compared with those of the WT mice (Figure 4(f)). Consistently, the production of the anti-inflammatory cytokines IL-10 and TGF-β was significantly increased in the colon of the *slamf7*^*-/-*^ mice (Figure S8c,d). To examine whether macrophages regulate mucus secretion from goblet cells in the absence of SLAMF7, which may in turn affect susceptibility to colitis, we intraperitoneally injected WT and *slamf7*^*-/-*^ mice with clodronate liposomes (neutral) to deplete colonic macrophages and then supplemented the treatment with DSS (Figure 4(g)). After clodronate liposome treatment, intestinal F4/80+ macrophages were depleted in both WT and *slamf7*^*-/-*^ mice (Figure S8e). Importantly, as a result of clodronate liposome treatment, we found a marked reduction in mucus layer thickness in clodronate liposome-treated *slamf7*^*-/-*^ mice (*slamf7*^*-/-*^-Clo), and there was no difference in mucus layer thickness otherwise (Figure 4(h-i)). These results demonstrate that intestinal macrophages from *slamf7*^*-/-*^ mice may contribute to excessive mucus production. Importantly, the *slamf7*^*-/-*^-Clo mice exhibited more severe colitis than the *slamf7*^*-/-*^ mice did following DSS treatment, while there were no differences in body weight, colon length, or mortality between the *slamf7*^−*/*-^-Clo and WT-Clo mice (Figure 4(j-l)). Moreover, we performed 16S rRNA sequencing of stool samples from WT and *slamf7*^*-/-*^ mice before and after treatment with clodronate liposomes. PCoA revealed that clodronate liposome treatment altered the composition of the microbiome in *slamf7*^*-/-*^ mice (*slamf7*^*-/-*^ vs. *slamf7*^*-/-*^-Clo), and the abundance of *Akkermansia muciniphila* decreased, but not significantly, in WT mice (Figure S8f,g). Therefore, polarized macrophages are involved in reducing the susceptibility of *slamf7*^*-/-*^ mice to DSS.

We next explored whether IL-10 and TGF-β contribute to excessive mucus production in *slamf7*^*-/-*^ mice. To test this hypothesis, we cocultured mouse intestinal stem cells with intestinal macrophages isolated from WT or *slamf7*^*-/-*^ mice and used IL-10 and TGF-β antibodies to block the cytokines IL-10 and TGF-β, respectively, in *slamf7*^*-/-*^ macrophages (*slamf7*^*-/-*^-Ab). We found no significant difference in enteroid number or enteroid area between the WT and *slamf7*^*-/-*^-Ab samples (Figure S9A-C). By using a Transwell insert system based on Caco-2 epithelial cells and intestinal macrophages isolated from WT mice and *slamf7*^*-/-*^ mice, we found that the expression of MUC2 in *slamf7*^*-/-*^-Ab and WT mice was comparable, but greater MUC2 production was observed in Caco-2 epithelial cells after coculture with macrophages from *slamf7*^*-/-*^ mice than after coculture with macrophages from WT mice (Figure S9d,e), suggesting that IL-10 and TGF-β regulate the amount of mucus in *slamf7*^*-/-*^ mice. Additionally, we treated *slamf7*^*-/-*^ mice with siRNAs targeting IL-10 and TGF-β (*slamf7*^*-/-*^ siRNA) (Figure S9f). The results confirmed that *in vivo* transfection with IL-10 and TGF-β siRNA decreased the abundance of IL-10 and TGF-β mRNAs in the colon of the *slamf7*^*-/-*^-siRNA mice (Figure S9g). We then challenged these *slamf7*^*-/-*^ mice with DSS and found that blocking IL-10 and TGF-β aggravated DSS-induced colitis, as the weight loss (Figure S9h), DAI score (Figure S9i), colon length (Figure S9j,k), and goblet cell number (Figure S9l,m) of these mice were similar to those of WT mice. These results support the idea that IL-10 and TGF-β may play a role in mitigating intestinal inflammation in *slamf7*^*-/-*^ mice.

### Upregulation of C1q induced by SLAMF7 depletion facilitates M2 macrophage polarization in response to DAMPs stimulation

**Figure 5.**
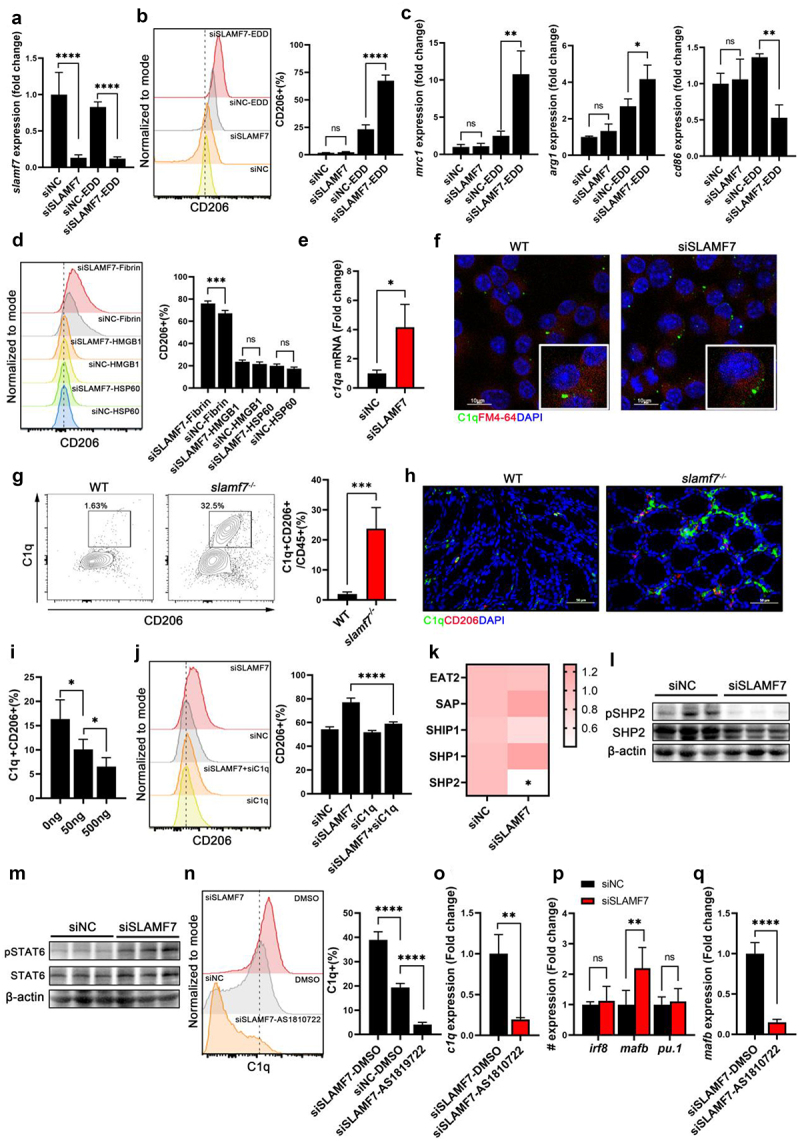
SLAMF7 suppressed fibrin-triggered M2 polarization.

When intestinal tissue is damaged, DAMPs, such as low doses of epithelium-derived DAMPs (EDDs), can promote the differentiation of M2 macrophages.^40^ Since we observed that the absence of SLAMF7 promotes M2 polarization in the colon without DSS treatment, we hypothesized that DAMPs may contribute to SLAMF7-mediated macrophage polarization. To test this hypothesis, an siRNA (siSLAMF7) was designed to reduce the expression of SLAMF7 (Figure 5(a)). RAW264.7 cells were transfected with SLAMF7 siRNA or negative control siRNA and then treated with EDDs. As shown in Figure 5(b,c), EDD treatment promoted M2 polarization, whereas SLAMF7 depletion significantly promoted M2 polarization in response to EDD stimulation. We then assessed the ability of different DAMPs to regulate macrophage polarization following SLAMF7 silencing. Extracellular (fibrin), cytosolic (HSP60), and nuclear (HMGB1) DAMPs were used to treat RAW264.7 cells. Interestingly, compared with the negative control, SLAMF7 knockdown increased the number of M2 macrophages induced by fibrin (Figure 5(d)), suggesting that fibrin regulates SLAMF7-mediated macrophage polarization. We further confirmed these phenotypes in bone marrow-derived macrophages (BMDMs) isolated from WT and *slamf7*^*-/-*^ mice (Figure S10A). Fibrin directly interacts with C1q or Mac-1 to facilitate various immune responses.^41,42^ Thus, we hypothesized that fibrin regulates M2 polarization following SLAMF7 knockdown via C1q or Mac-1. We then used siRNA to deplete SLAMF7 in RAW264.7 cells and detected the expression of C1q and Mac-1. No significant changes in Mac-1 mRNA levels were detected between the siNC and siSLAMF7 groups (Figure S10b), whereas C1q was significantly increased in SLAMF7-knockdown cells (Figure 5(e)). Confocal microscopy imaging of FITC-stained C1q revealed that C1q was increasingly expressed on the surface of RAW264.7 cells with SLAMF7 knockdown. Consistently, a dramatically greater proportion of C1q-expressing CD206+ macrophages was observed in the colons of the *slamf7*^*-/-*^ mice than in those of the WT mice (Figure 5(f-h)). To determine whether SLAMF7 activation had the opposite effect on M2 macrophages, RAW264.7 cells were treated with recombinant SLAMF7 protein (rm-SLAMF7). As expected, SLAMF7 activation partially suppressed macrophage M2 polarization in response to fibrin stimulation (Figure 5(i)). To explore the impact of C1q on SLAMF7-mediated macrophage polarization, RAW264.7 cells were transfected with siSLAMF7 and siC1q and then treated with fibrin. qPCR revealed that the expression of SLAMF7 and C1q was significantly decreased by siRNA (Figure S10c). We found that the number of M2 macrophages significantly decreased following siSLAMF7 and siC1q depletion, suggesting that C1q is required for M2 macrophage induction by SLAMF7 knockdown (Figure 5(j)). These results demonstrate that SLAMF7 depletion upregulates C1q expression, priming macrophages to polarize toward the M2 phenotype prior to fibrin stimulation.

RAW264.7 cells were treated with control siRNA or siRNA targeting SLAMF7 and then exposed to epithelial-derived damps (EDDs, *n* = 3/group). (a) SLAMF7 expression was quantified relative to that in RAW264.7 cells treated with control siRNA by RT‒PCR. Macrophage subtype-specific markers were analyzed by flow cytometry (b) and qPCR (c). Purified DAMPs were used to stimulate RAW264.7 cells. (d) CD206+ macrophages were analyzed by flow cytometry (*n* = 5/group). (e) qPCR was used to quantify fibrin receptor expression in macrophages (*n* = 3/group). (f) Representative confocal microscopy images of RAW264.7 cells stained with an anti-C1q antibody and membrane dye are shown; scale bar, 10 μm. Flow cytometry (g) and immunofluorescence staining (h) were used to identify colonic C1q+CD206+ macrophages in WT and *slamf7*^*-/-*^ mice (*n* = 5/group). Recombinant SLAMF7 was used to activate SLAMF7 in RAW264.7 cells (*n* = 5/group). Flow cytometry was used to identify C1q+CD206+ macrophages (i). RAW264.7 cells were treated with siRNA targeting *c1q* or SLAMF7 and then stimulated with fibrin (*n* = 3/group). The expression of the macrophage subtype-specific marker CD206 was analyzed by flow cytometry (j). (k) qPCR was used to quantify SLAMF7 adaptors in macrophages (*n* = 5/group). Western blotting was used to detect phosphorylated SHP2/SHP2 (l) and phosphorylated STAT6/STAT6 (m) in RAW264.7 cells. RAW264.7 cells were treated with a STAT6 inhibitor (AS1819722, *n* = 5/group). Macrophage subtype-specific markers C1q were analyzed by flow cytometry (n) and qPCR (o). (p) qPCR was used to quantify the expression of C1q transcription factors in SLAMF7-silenced RAW264.7 cells. (q) qPCR was used to quantify *mafb* expression in SLAMF7-silenced RAW264.7 cells after STAT6 inhibitor treatment (*n* = 3/group). The data are presented as the means ± SEMs. *p < 0.05; **p < 0.01; ***p < 0.001; ****p < 0.0001.

SLAMF7 recruits SH2 domain – containing molecules to activate downstream signals. To explore the mechanism by which SLAMF7 regulates *c1q* expression, we used fibrin to examine the effect of SLAMF7 on macrophage polarization. First, we detected the expression of cytoplasmic adapter proteins. Interestingly, the expression levels of Eat-2, SHP1 and Sap did not change following SLAMF7 silencing, whereas SHP2 expression was significantly decreased in SLAMF7-deficient macrophages (Figure 5(k) and Figure S10d). We confirmed that SLAMF7 was able to interact with SHP2 directly by IP analysis (Figure S10e). To further confirm that SLAMF7 regulates macrophages via SHP2, we transfected RAW264.7 cells with siSLAMF7 and SHP2 plasmids. Conversely, the overexpression of SHP2 rescued the M2 polarization of SLAMF7-silenced macrophages during fibrin treatment (Figure S10f). A previous study reported that SLAMF7 can phosphorylate cytoplasmic adapter proteins,^16^ so we measured the levels of SHP2 and phosphorylated SHP2 (p-SHP2), the active form of SHP2, in RAW264.7 cells with or without siSLAMF7. As shown in Figure 5l, SHP-2 and p-SHP2 levels were decreased in SLAMF7-knockdown cells. Previous studies have shown that SHP2 inactivation augments JAK1/STAT6 signaling through its phosphatase activity, leading to an IL-4–mediated shift toward M2 polarization.^43^ Indeed, the protein expression of phosphorylated STAT6 (p-STAT6), the active form of STAT6, significantly increased after SLAMF7 knockdown (Figure 5m). To confirm the involvement of STAT6 in SLAMF7 knockdown-mediated M2 polarization, the STAT6 inhibitor AS1810722 was used to inhibit STAT6 phosphorylation. Consistently, inhibitor treatment significantly decreased *c1q* expression in SLAMF7-knockdown cells (Figure 5(n,o)). These data illustrate that SLAMF7 silencing reduces SHP2, which may contribute to activation of the STAT6 pathway – a signaling axis linked to M2 polarization.

The transcription factors IRF8, MafB and PU.1 are reported to directly bind to the *c1q* promoter. We therefore determined the mRNA levels of these transcription factors in RAW264.7 cells following SLAMF7 silencing (Figure 5(p)). No significant changes in IRF8 or PU.1 mRNA levels were observed in SLAMF7-knockdown cells compared with siNC cells, whereas MafB was significantly increased after SLAMF7 depletion. Since the transcription factor MafB, a direct transcriptional target of STAT6, promotes M2 polarization,^44^ we hypothesized that, in SLAMF7-knockdown cells, upregulated *mafB* facilitated *c1q* expression. Indeed, we found that inhibition of STAT6 by its inhibitor significantly decreased the expression of C1q (Figure 5(q)).

Together, our findings establish that SLAMF7 depletion in macrophages is correlated with C1q upregulation, potentially mediated by the STAT6-MafB axis, resulting in a bias toward the M2 phenotype under DAMPs stimulation.

### SLAMF7 as a potential therapeutic target in IBD

**Figure 6.**
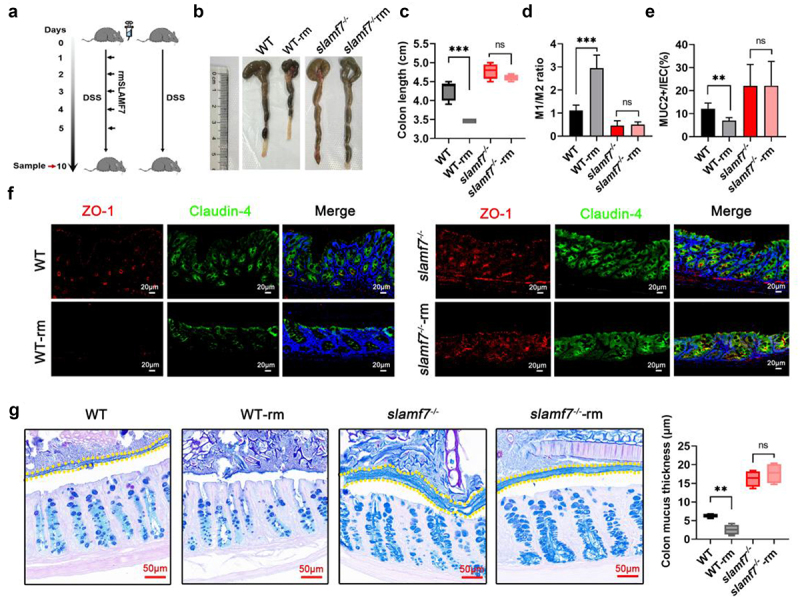
SLAMF7 engagement increased susceptibility to colitis.

Given the critical role of SLAMF7 in the pathogenesis of IBD, we postulated its potential as a therapeutic target. To test this hypothesis, we first investigated whether SLAMF7 engagement aggravated colitis. SLAMF7 is a self-ligand receptor that is activated by recombinant SLAMF7 protein (rm-SLAMF7) or its monoclonal antibody. We next treated WT and *slamf7*^*-/-*^ mice with recombinant SLAMF7 and DSS (Figure 6(a)). After DSS treatment, the colon length was shorter in the rm-SLAMF7-treated WT mice (WT-rm) than in the WT mice (Figure 6(b,c)), although slight differences in body weight and the DAI score were detected between these two groups of mice (Figure S11a,b). As expected, there was no difference in colon length or body weight between *slamf7*^*-/-*^ and *slamf7*^*-/-*^-rm mice due to SLAMF7 deficiency. Consistent with our observation of a reduced M1/M2 ratio in the absence of SLAMF7, SLAMF7 activation by rm-SLAMF7 increased the M1/M2 ratio (Figure 6(d)), which in turn decreased the number of goblet cells in the colon tissue of WT-rm mice (Figure 6(e)), indicating that SLAMF7 may govern the switching of M1/M2-like macrophages. To determine whether SLAMF7 activation impaired intestinal barrier integrity, we measured the levels of the tight junction proteins ZO-1 and claudin-4 and detected lower levels of ZO-1 and claudin-4 in the colon tissues of WT-rm mice (Figure 6(f)). Importantly, rm-SLAMF7 treatment partially reduced mucus production in WT-rm mice (Figure 6(g)). These results indicate that SLAMF7 activation moderately enhances colitis susceptibility in WT mice.

(a) The intestinal administration strategy for recombinant SLAMF7 is shown. (b-c) After intestinal administration of rm-SLAMF7 and 2% DSS water, the mouse colon (*n* = 5/group) was separated (b), and the colon length was measured (c). (d-e) Flow cytometry analysis showing the M1/M2 ratio (d) and frequency of MUC2+ cells (e) in the colon. (f) Immunofluorescence staining of tight junction proteins in mouse colon tissue sections was performed; scale bars: 20 μm. (g) AB‒PAS staining of the colon mucous layer was performed; scale bars: 50 μm. The data are presented as the means ± SEMs. ns, not significant; *p < 0.05; **p < 0.01; ***p < 0.001.

**Figure 7.**
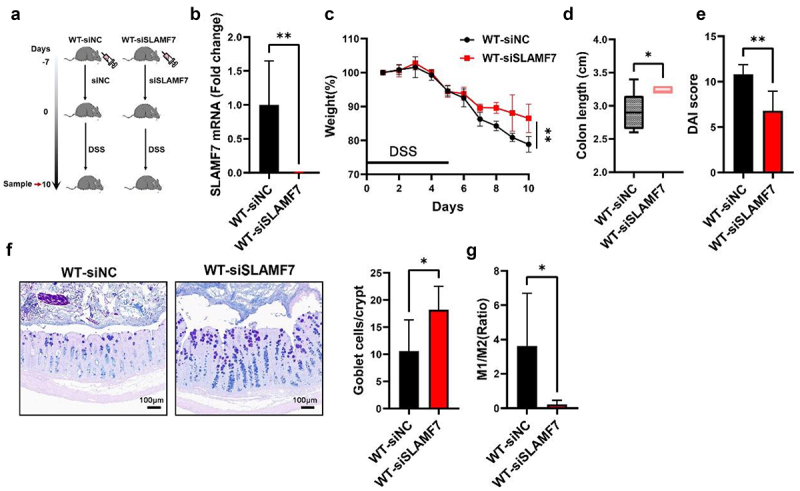
Silencing SLAMF7 in the intestine relieved inflammation.

Next, we investigated whether SLAMF7 knockdown could promote intestinal resolution. SLAMF7-targeting siRNA (WT-siSLAMF7) was administered to the intestines of the mice (Figure 7(a)). The results verified that *in vivo* transfection with SLAMF7 siRNA reduced the levels of SLAMF7 mRNA in the mouse colon (Figure 7(b)). Subsequently, these mice were exposed to DSS, and it was observed that SLAMF7 silencing ameliorated DSS-induced colitis, as evidenced by reduced weight loss (Figure 7(c)), increased colon length (Figure 7(d)), a lower DAI score (Figure 7(e)), and an increased number of goblet cells (Figure 7(f)) compared to those of WT-siNC mice. Additionally, the reduction in SLAMF7 expression facilitated the polarization of CD206+ macrophages (Figure 7(g)).

The strategy for the intestinal delivery of siSLAMF7 is shown. (b) SLAMF7 mRNA expression in the mouse intestine after siSLAMF7 and DSS treatment (*n* = 5/group). (c) After intestinal administration of siSLAMF7 and 2% DSS-containing water, the mouse colon was collected (d) and the DAI score was measured (e). (f) AB‒PAS staining of goblet cells in colon tissues; scale bars: 100 μm. (g) Flow cytometry analysis was used to determine the M1/M2 ratio. The data are presented as the means ± SEMs. *p < 0.05; **p < 0.01.

Overall, these findings underscore the pivotal role of SLAMF7 in directing macrophage polarization toward an inflammatory phenotype, thereby providing new insights into the mechanisms underlying the pathogenesis of IBD.

## Discussion

Dysregulation of the balance of M1 and M2 macrophages is a hallmark of chronic inflammatory conditions such as IBD. This study demonstrates that SLAMF7 critically regulates intestinal mucus layer formation by influencing goblet cell proliferation, a process mediated by macrophage polarization. SLAMF7 deficiency promoted tissue protection during DSS-induced intestinal damage by altering the gut microbiome composition and enhancing mucosal barrier integrity. SLAMF7 deficiency promoted the M2 polarization of macrophages, resulting in the formation of a thicker mucus layer, which in turn increased the abundance of the protective commensal bacterium *Akkermansia muciniphila*. Mechanistically, SLAMF7 depletion increased C1q expression through activation of the STAT6-MafB pathway, thus facilitating M2 polarization in response to DAMP stimulation. These findings highlight a previously unrecognized role of SLAMF7 in regulating mucosal immunity and the composition of the intestinal microbiota. This discovery highlights the potential for novel strategies to treat diseases rooted in immune – microbiota dysregulation. Prioritizing translational studies will clarify the therapeutic potential of SLAMF7 while addressing safety and efficacy challenges.

Previous studies have shown that SLAMF7 suppresses the inflammatory response in murine models of sepsis and keratitis and that SLAMF7-knockout mice exhibit more severe tissue damage than do WT mice.^16,17^ However, in other contexts, activation of the SLAMF7 receptor in macrophages leads to rapid and extensive production of inflammatory cytokines and chemokines. Importantly, the level of SLAMF7 and its activation signature are increased in IBD patients, suggesting that SLAMF7 contributes to macrophage-driven inflammation in IBD.^18^ Similar to our observations, previous studies have shown that several SLAMF7 family proteins promote the development of colitis, especially in mice without an adaptive immune system (Rag-1-knockout mice).^45,46^ For example, the deletion of SLAMF6 in Rag-1-knockout mice leads to reduced susceptibility to colitis after oral infection with the intestinal pathogen *Citrobacter rodentium* compared with that in Rag-1-deficient mice, but this protective phenotype was not observed in mice that lacked only SLAMF6.^47,48^ In this work, we extended these studies to identify the previously unrecognized functions of SLAMF7 in regulating intestinal inflammation and tissue homeostasis. Compared with that in WT mice, DSS-induced colitis was markedly reduced in mice without SLAMF7.

Given that SLAMF7-deficient mice exhibited dramatically reduced susceptibility to colitis compared with WT mice and that SLAMF7 activated by recombinant SLAMF7 exacerbates colitis, we expect that blockade of SLAMF7 would relieve intestinal inflammation and have important therapeutic implications. This differs from findings in cancer immunotherapy, where SLAMF7 has emerged as an attractive therapeutic target owing to its ability to specifically activate NK cells within tumors and promote immunogenic cell death.^49^ The function of SLAMF7 in both inflammation and cancer immunotherapy depends on its specific environment and molecular mechanisms. For example, in renal cell carcinoma, activation of the self-ligand SLAMF7 immune receptor on T cells induces STAT1 and STAT3 phosphorylation and the expression of multiple inhibitory receptors and transcription factors associated with T-cell exhaustion. Mice lacking SLAMF7 show restricted growth of tumors.^50^ In addition, macrophages selectively phagocytose hematopoietic tumor cells in response to SIRPα–CD47 checkpoint blockade in a SLAMF7-dependent manner.^51^ In multiple myeloma, SLAMF7 is highly expressed on multiple myeloma cells but has limited expression in a subset of hematopoietic cells among normal tissues, making it a rational target for cancer therapy.^52^ The SLAMF7 antibody elotuzumab has been approved for the treatment of patients with multiple myeloma. This antibody binds to SLAMF7 on myeloma cells, simultaneously activating NK cells and macrophages through the SLAMF7-EAT2 interaction and stimulating the FYN/Syk-PI3K-Akt/mTOR signaling pathway. This process increases NK cell-mediated cytotoxicity and triggers the release of IFN-γ, TNF-α, and granzymes, leading to the targeted destruction of myeloma cells through apoptosis.^53^ Our findings suggest that SLAMF7 activation drives intestinal inflammation by decreasing C1q+ M2-like macrophages and downregulating their associated cytokines, IL-10 and TGF-β, via the SLAMF7-SHP2-STAT6 pathway in the gut. Consistently, a very recent study revealed that in hepatocellular carcinoma (HCC), SLAMF7 upregulation was observed in immunotherapy-responsive HCC tumors, with responding patients exhibiting elevated serum SLAMF7 levels. Mechanistically, SLAMF7 deficiency in HCC cells triggers CCL2 upregulation, which subsequently promotes immunosuppressive macrophage polarization. These data support the idea that the immune microenvironment may contribute to SLAMF7-mediated macrophage polarization. Notably, common adverse reactions to elotuzumab include diarrhea, upper respiratory tract infection, and decreased appetite.^54^ Thus, elotuzumab treatment likely impacts intestinal homeostasis. Future research should investigate whether elotuzumab treatment exacerbates intestinal damage and inflammation, particularly in IBD patients. With the exception of IBD patients, the upregulated expression of SLAMF7 was detected in patients with acute and chronic inflammation,^18^ such as those with rheumatoid arthritis and COVID-19 pneumonia, indicating the extensive role of SLAMF7 in regulating the inflammatory response. Although we propose SLAMF7 as a potential therapeutic target in IBD, interference with the SLAMF7 signaling pathway through targeted drugs may increase infection risk. As SLAMF7 is highly expressed in multiple myeloma cells and macrophages under conditions of bacterial infection,^16,53^ suppression of SLAMF7 may contribute to treatment failure in multiple myeloma patients and exacerbate hyperinflammatory responses to systemic bacterial infections. Consequently, the development of intestinal-specific targeting of this receptor may offer a promising strategy to suppress excessive inflammatory responses.

Our data demonstrated that EDDs, particularly stimulation with fibrin, can promote M2 skewing following SLAMF7 silencing. Moreover, SLAMF7 inactivation increased the number of M2 macrophages at a steady state, suggesting that the colon microenvironment in SLAMF7 deficiency mice facilitates M2 polarization. Consistent with our results, previous studies have shown that *slamf7*^−/−^ mice without an adaptive immune system also have fewer M1 macrophages in the inflamed colon than do control mice.^46^ Intestinal epithelial cells have an average lifespan of one day,^55^ and dying cells release various DAMPs, such as fibrin. These DAMPs can trigger further epithelial cell death. Notably, SLAMF7 knockdown increased the expression of the fibrin receptor C1q, enhancing the ability of macrophages to sense fibrin. Studies have demonstrated that fibrin stimulation promotes M2 polarization.^56,57^ Our study revealed that C1q is required for M2 macrophage induction following SLAMF7 knockdown. Recently, C1q-expressing macrophages were found to express C1QB, APOE, and CD163, which are key phenotypic markers for M2 macrophages.^58^ These data support our notion that the increase in C1q induced by SLAMF7 depletion facilitates M2 macrophage polarization in response to DAMP stimulation. More importantly, our results demonstrate that SLAMF7 depletion increases C1q levels by activation of the STAT6-mafB pathway. It has been reported that SLAMF family protein self-engagement transduces positive signals by recruiting a family of adaptors, including SLAM-associated protein (SAP), SHP-1 and/or SHP-2.^59^ SLAMF7 self-engagement activates the SHP-2 signaling pathway in myeloma cells.^60^ Although we observed that SLAMF7 depletion decreased SHP-2 in macrophages, further studies are necessary to elucidate the molecular mechanism underlying this phenomenon.

Finally, we found that macrophages isolated from *slamf7*^*-/*-^ mice promoted intestinal stem cell proliferation and increased mucus production in Caco-2 epithelial cells. Wnt secretion by CD206+ macrophages promotes the proliferation of mesenchymal niche cells.^61^ Moreover, several anti-inflammatory cytokines, such as IL-10 and TGF-β, improve goblet cell function to increase mucus secretion.^62,63^ As expected, increased numbers of CD206+ macrophages were sufficient to form a thicker mucus layer in SLAMF7-knockout mice.^64^ Consistent with these findings, we found that loss of SLAMF7 increased MUC2 secretion and altered the gut microbiome, specifically by promoting the secretion of mucus-utilizing bacteria such as *Akkermansia muciniphila*. Several studies have convincingly shown that *Akkermansia muciniphila* has protective effects against IBD.^65^ *Akkermansia muciniphila* degrades mucin glycoproteins through its enzymatic activity and stimulates goblet cells to increase mucus production, thereby facilitating mucous layer renewal.^66^ In addition, *Akkermansia muciniphila* secretes threonyl-tRNA synthetase to restore macrophage homeostasis, increases IL-10 levels, and attenuates colitis in mice.^36^ Our data reveal that SLAMF7 deficiency increases MUC2 secretion and increases the abundance of *Akkermansia muciniphila*, suggesting a protective role of *Akkermansia muciniphila* in *slamf7*^*-/-*^ mice after DSS treatment. Future research on the protective role of *Akkermansia muciniphila* in *slamf7*^*-/-*^ mice would be highly valuable. In addition to *Akkermansia muciniphila*, *slamf7*^−/−^ mice exhibit an increase in *Christensenellaceae R_7* and a reduction in pathogenic *Enterococcus/Streptococcus*. IBD patients show decreased *Christensenellaceae* abundance, suggesting its homeostatic role.^67^ Moreover, the mucus layer supports beneficial microbes that enhance colonization resistance through niche competition and metabolites, such as tryptophan metabolites and short-chain fatty acids (SCFAs). We observed increased tryptophan metabolites in the gut of SLAMF7-deficient mice. Further investigations are needed to determine whether the gut microbiota in *slamf7*^−/−^ mice enhances resistance against pathogenic organism colonization. These microbial metabolites contribute to the reinforcement of the mucus layer, strengthening its protective function and further preventing pathogenic colonization.^64,65,68^

In conclusion, our findings demonstrate that SLAMF7 critically regulates macrophage polarization, influencing mucus secretion and gut microbial homeostasis. These results establish SLAMF7 as a therapeutically actionable target in IBD, where dysregulated macrophage responses drive chronic inflammation. By restoring the balance between proinflammatory and anti-inflammatory pathways, targeted modulation of SLAMF7 signaling offers a clinically viable strategy to disrupt the cycle of mucosal damage and inflammation in IBD patients. This work not only identifies SLAMF7 as a central player in IBD pathogenesis but also provides a foundational framework for developing macrophage-centric therapies to improve long-term outcomes in individuals with refractory or relapsing disease.

## Supplementary Material

Supplemental Material

Supplemental Table 1.xlsx
