# Supplementary material for: SLAMF7 regulates goblet cell mucus production and negatively impacts gut homeostasis and commensalism
Source: Gut Microbes. 2025 Jul 11;17(1):2527857. doi: 10.1080/19490976.2025.2527857 (PMC12258210; doi:10.1080/19490976.2025.2527857)
Supplement: Supplemental Material [file KGMI_A_2527857_SM4406.docx]

**Supplemental information titles and legends**

**Figure S1. Immunofluorescence staining ZO-1 protein positive control.**

1. Immunofluorescence staining for ZO-1 in colonic sections from DSS-untreated mice. Scale bar, 20 μm.(B) Western blotting analysis of ZO-1 protein expression in colonic tissues after DSS treatment (n=6/group).

The data are presented as the means ± SEMs. ***p* < 0.01.

**
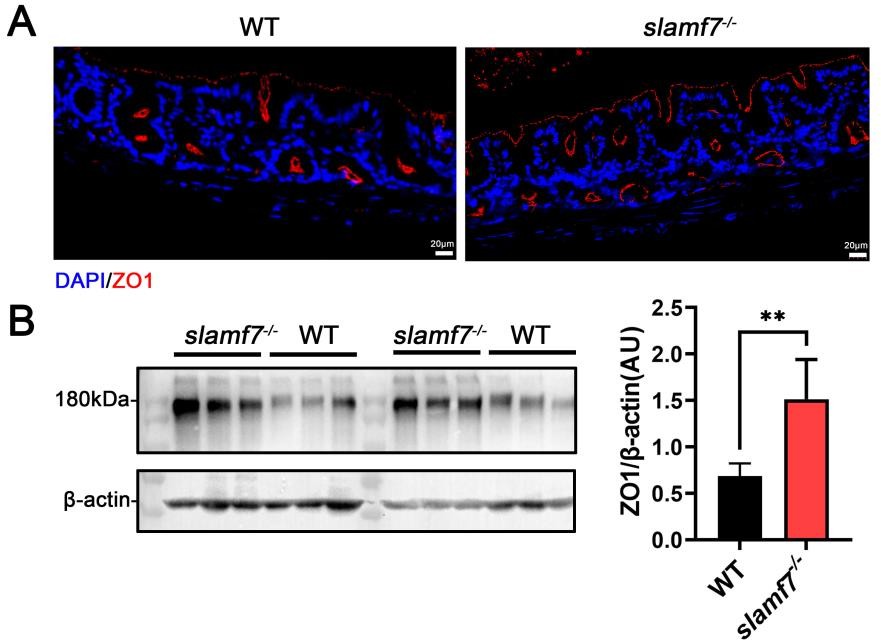
**

**Figure S2. Mouse colon cells were subclustered according to marker genes.**

(A) Heatmap showing subtype-specific genes. The expression of marker genes (rows) across cell subsets (columns) ordered by cell lineage relationships (top, color legend) is shown.

(B) Bubble plot showing marker genes expressed in WT and *slamf7^-/-^* mice. Pct: Percent of cells with expression of genes in clusters.


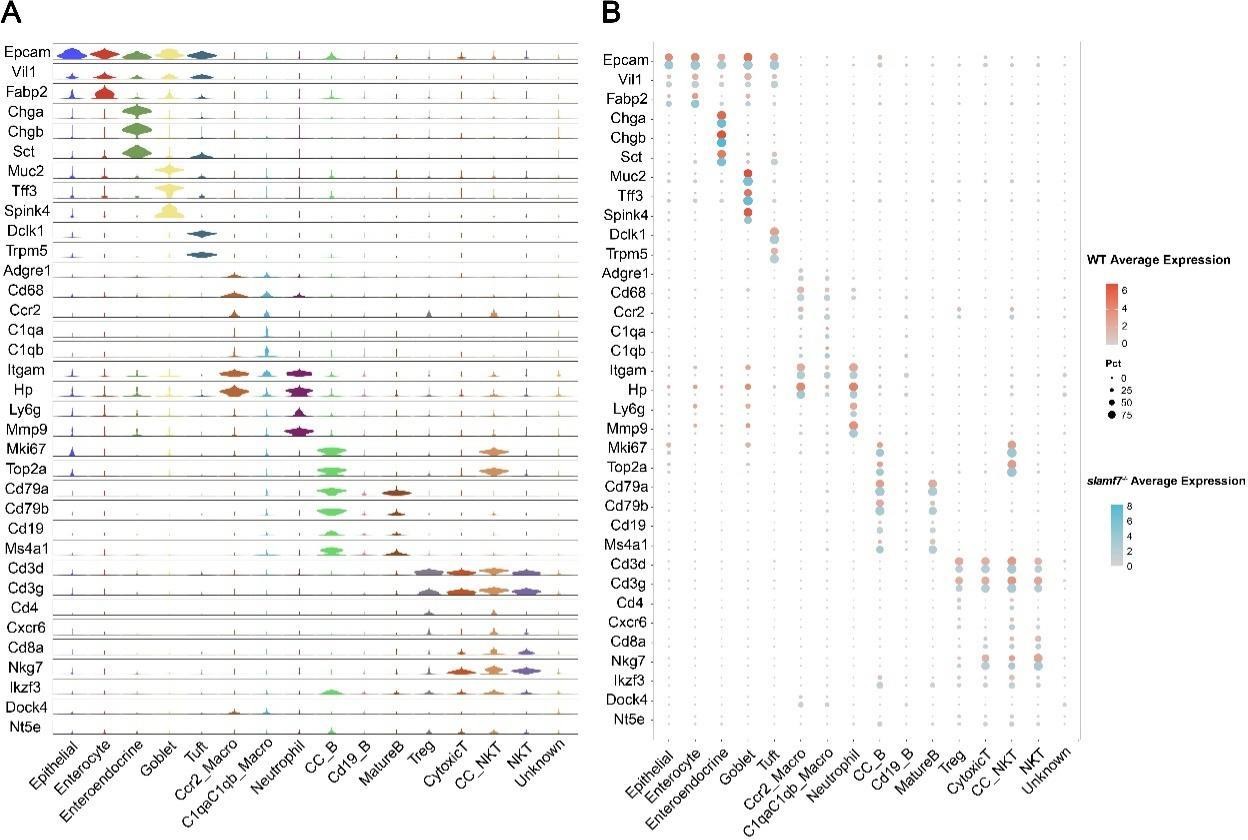


**Figure S3. Composition and functions of lymphocytes in the colon of WT and *slamf7^-/-^* mice.**

(A) t-SNE plots displaying T cells and NK cells in the colon of WT and *slamf7^-/-^* mice.

(B) t-SNE plots displaying B cells in the colon of WT and *slamf7^-/-^* mice.

(C) GSEA of the lymphocyte pathway. NES, normalized enrichment score.

(D) The frequency of colonic lymphocytes was determined by flow cytometry.

(E) Percent of cells with expression of the J chain in the CC-B cluster.

(F) KEGG enrichment analysis of CC_B.

The data are presented as the means ± SEMs. ns, not significant, **p* < 0.05; ***p* < 0.01; ****p* < 0.001.


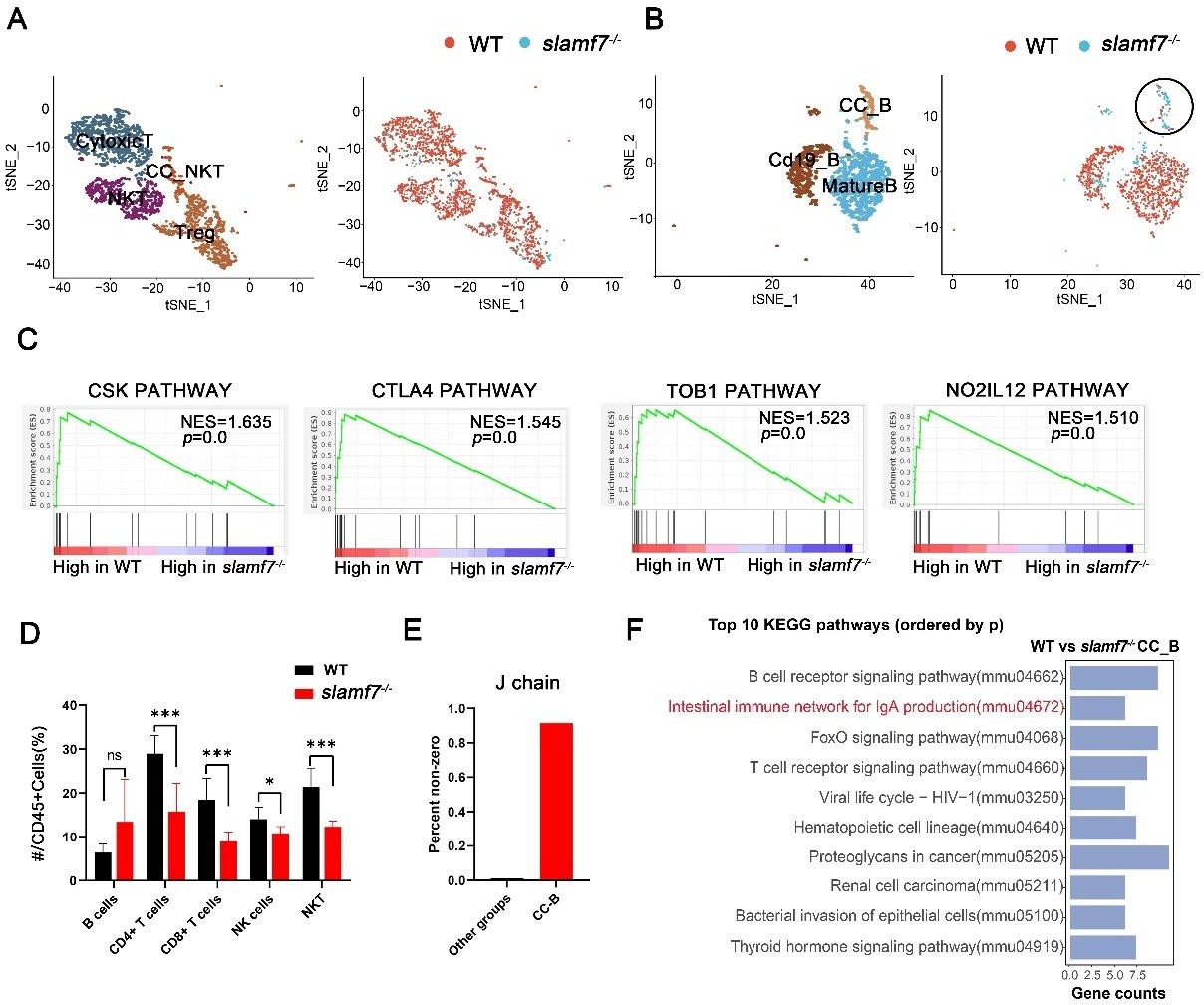


**Figure S4. Composition and function of myeloid cells in the colons of WT and *slamf7^-/-^*mice.**

(A) t-SNE plots displaying myeloid cells in the colons of WT and *slamf7^-/-^* mice.

(B) Violin plots of gene expression in C1qaC1qb macrophage clusters (upper). Percent of cells with expression of genes in C1qaC1qb macrophage clusters (below).

(C) Differential gene expression for C1qaC1qb macrophages versus other clusters.

(D) GO enrichment analysis of C1qaC1qb macrophage clusters and other clusters.


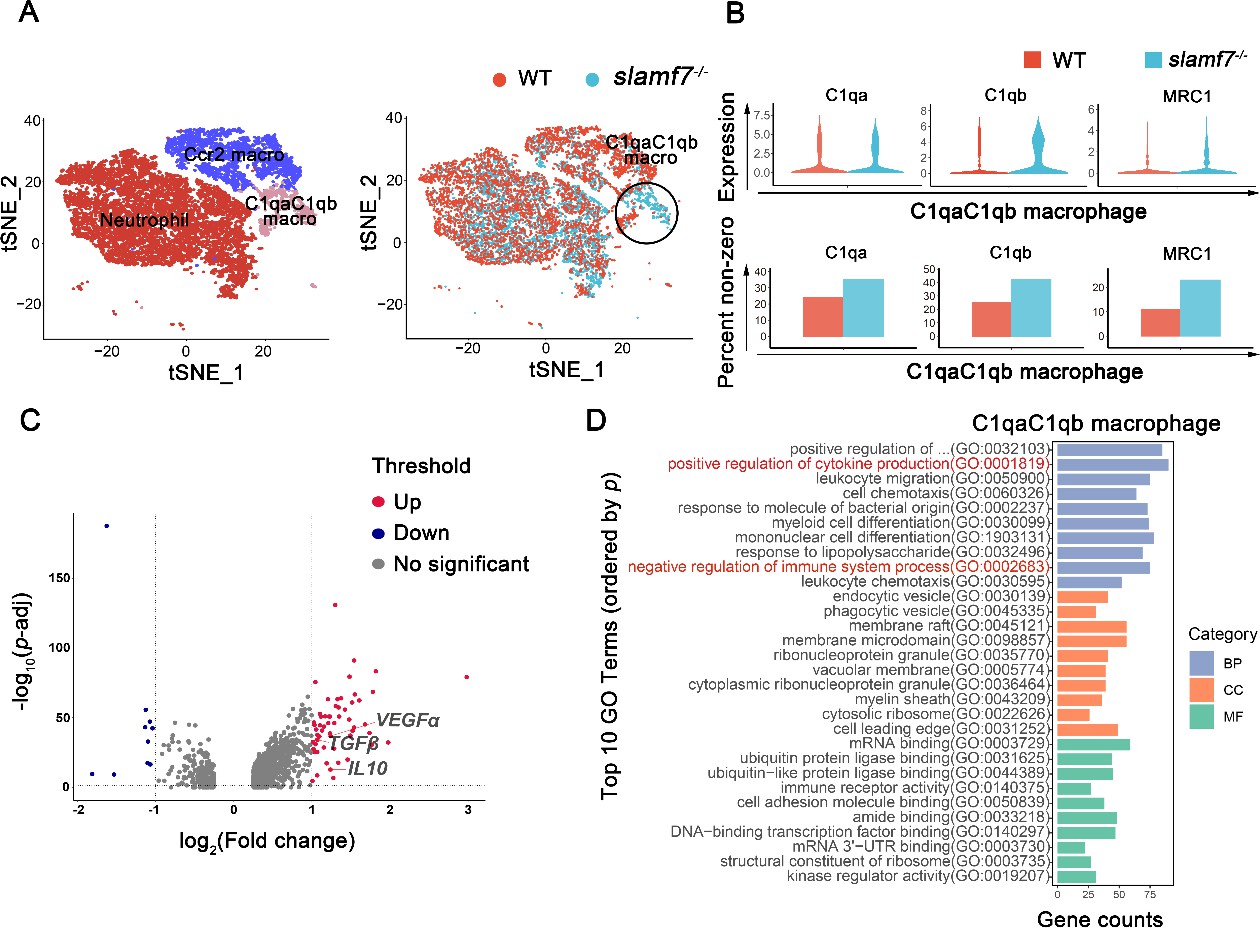


**Figure S5. Composition and function of the epithelium in WT and *slamf7^-/-^* mouse colons.**

(A) t-SNE plots displaying epithelial cells in the colons of WT and *slamf7^-/-^* mice.

(B) GSEA of the IGF-1 receptor and longevity pathway.

(C) GO analysis of goblet cells in the colon of WT and *slamf7^-/-^* mice.


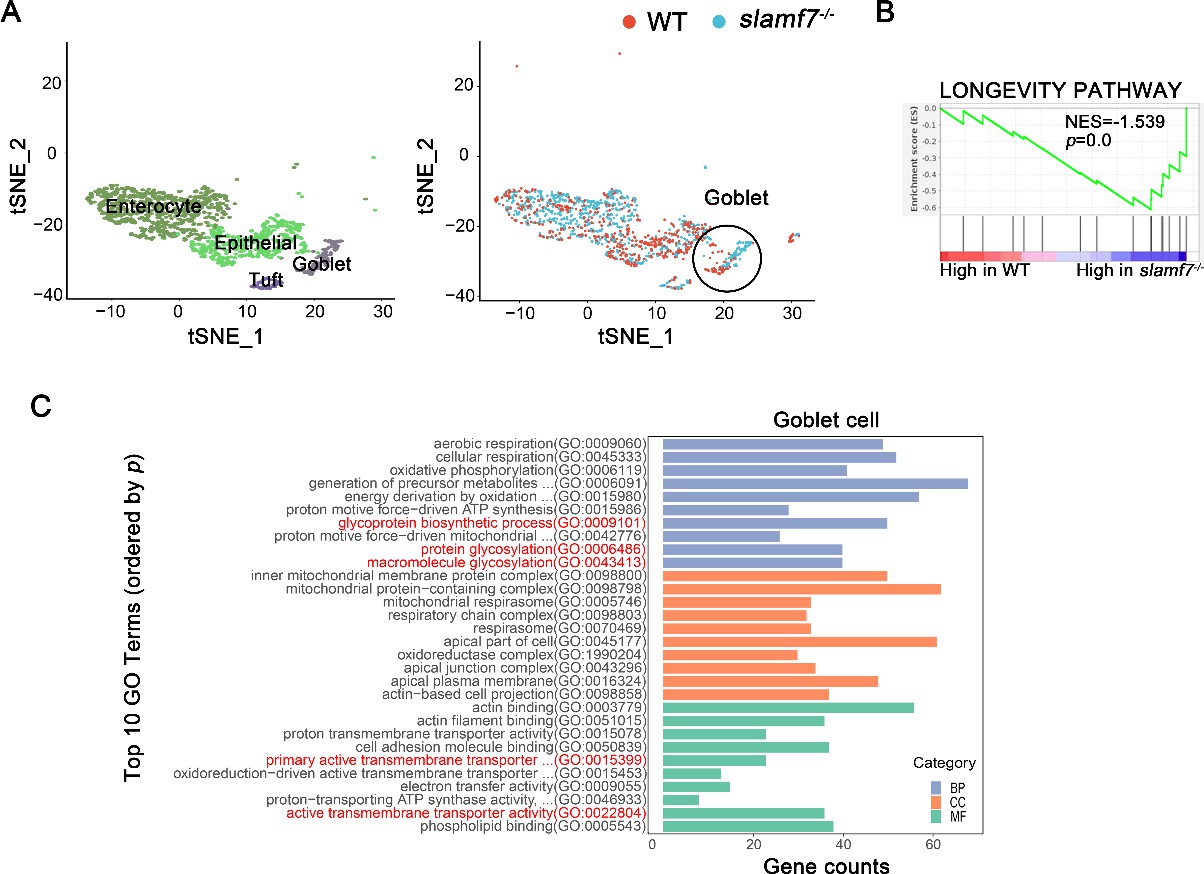


**Figure S6. The gut microbiome and metabolite profiles of stool samples from *slamf7^-/-^* and WT mice at steady state.**

(A) Circular Sankey diagram showing the overlap of the differential fecal microbiota enriched in *slamf7^-/-^* mice (n=14) in the IBD model relative to WT (n=14) and the differential fecal microbiota enriched relative to healthy individuals (con, n=72) in IBD patients (UC, n=58) downloaded from the NCBI gene database.

(B) PCoA of the fecal microbiota based on the Bray‒Curtis distance in untreated WT and *slamf7^-/-^* (n=21) mice in four batches.

(C) The raw intensity of tryptophan metabolites was measured.

(D) Bubble plot showing the top 10 enriched KEGG pathways. A differential abundance (DA) score > 0 indicates enrichment in *slamf7^-/-^* mice (n=5/group).

The data are presented as the means ± SEMs. ns, not significant, **p* < 0.05; ***p* < 0.01.


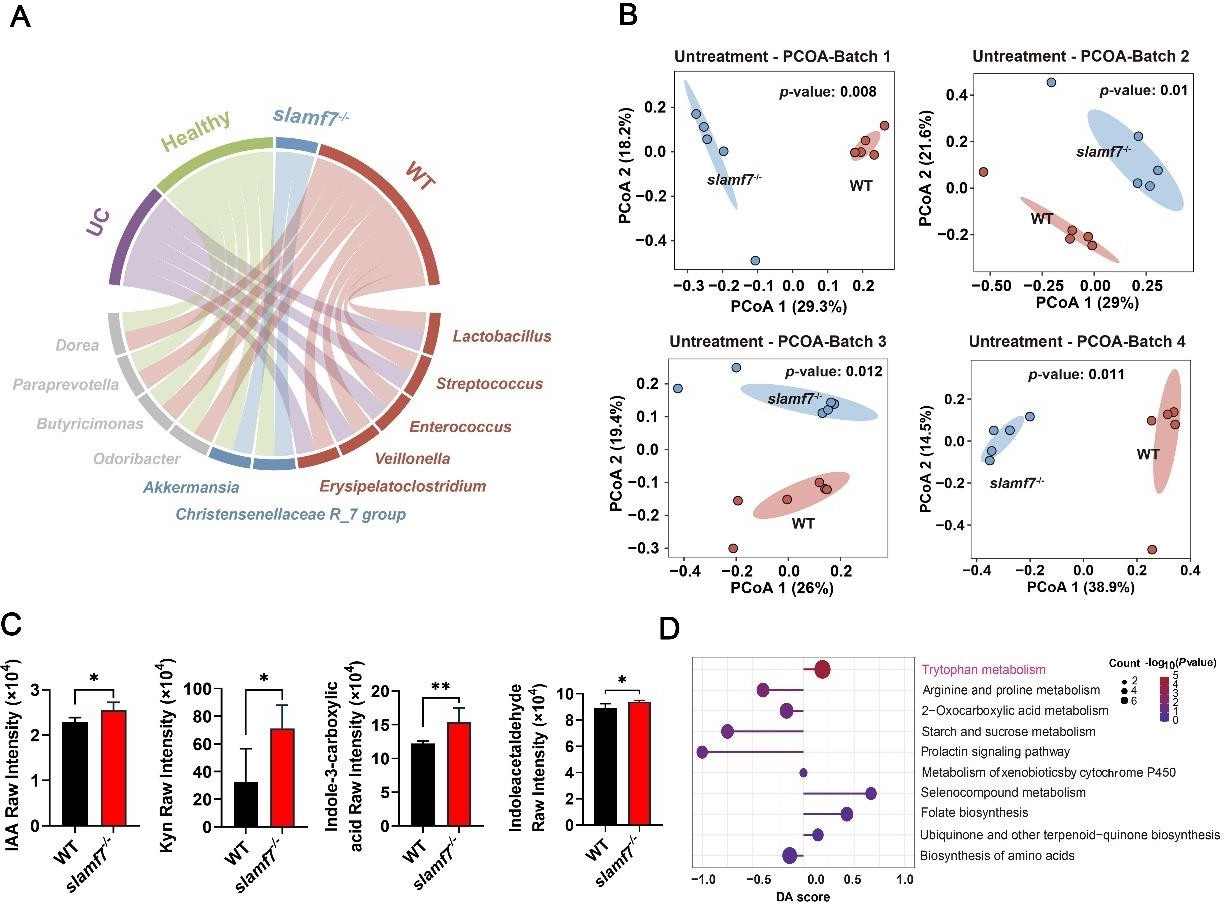


**Figure S7. SLAMF7 deficiency relieved mice colitis in a microbiota-dependent manner**

(A) Schematic diagram of ABX treatment for microbiota depletion in the mouse model. (n=5/group).

(B) Microbiome dissimilarity values were determined on the basis of the Bray‒Curtis distances of WT, *slamf7^-/-^* mice (n=5/group), and ABX-treated WT and *slamf7^-/-^* mice (n=5/group).

(C-E) Body weight (C), colon length (D), and DAI score (E) of the mice in (A).

(F) Abundance of *Akkermansia muciniphila* in the fecal microbiota of WT and *slamf7^-/-^* in cohoused model. (G) Abundance of *Akkermansia muciniphila* in the fecal microbiota of WT and *slamf7^-/-^* mice in the FMT model.

(H) PCoA of the fecal microbiota based on the Bray‒Curtis distance in WT and *slamf7*^-/-^ mice in the microbiome rebuilding model (n=5/group).

(I) Relative abundance of bacterial genera in the fecal microbiota of WT and *slamf7*^-/-^ mice in the microbiome rebuilding model.

(J) The relative abundance of *Akkermansia muciniphila* was determined at days 7 and 21.


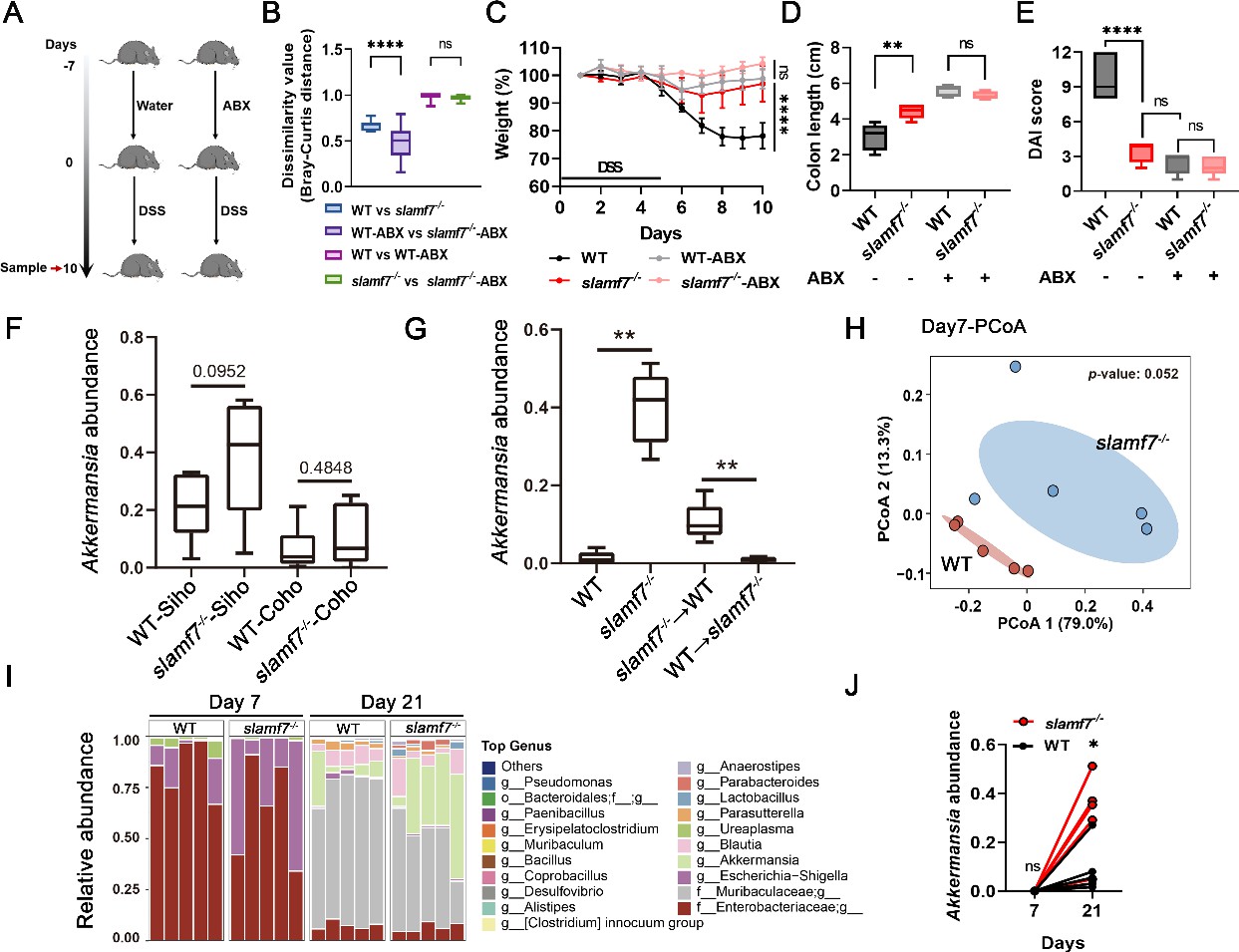


**Figure S8. Colonic macrophages isolated from *slamf7^-/-^* mice promoted goblet cell proliferation and mucin expansion.**

(A) Flow cytometric analysis of the particles in Figure 5A. Particle quantity in a well (B).

(C) qRT‒PCR analysis of cytokines in colon tissue.

(D) ELISA quantification of cytokines in colon tissue at steady state.

(E) Flow cytometry analysis of the frequency of macrophages in the mouse colon (n=5/group) after intraperitoneal injection of clodronate liposomes (neutral).

(F) PCoA of the fecal microbiota based on the Bray‒Curtis distance in WT and *slamf7*^-/-^ mice after clodronate liposome (neutral) treatment (WT: n=6, *slamf7*^-/-^: n=5, WT-Clo: n=6, *slamf7*^-/-^-Clo: n=5).

(G) Abundance of *Akkermansia muciniphila* in the fecal microbiota of WT and *slamf7^-/-^* mice before and after intraperitoneal injection of clodronate liposomes (neutral).

The data are presented as the means ± SEMs. ns, not significant, **p* < 0.05; ***p* < 0.01; ****p* < 0.001.


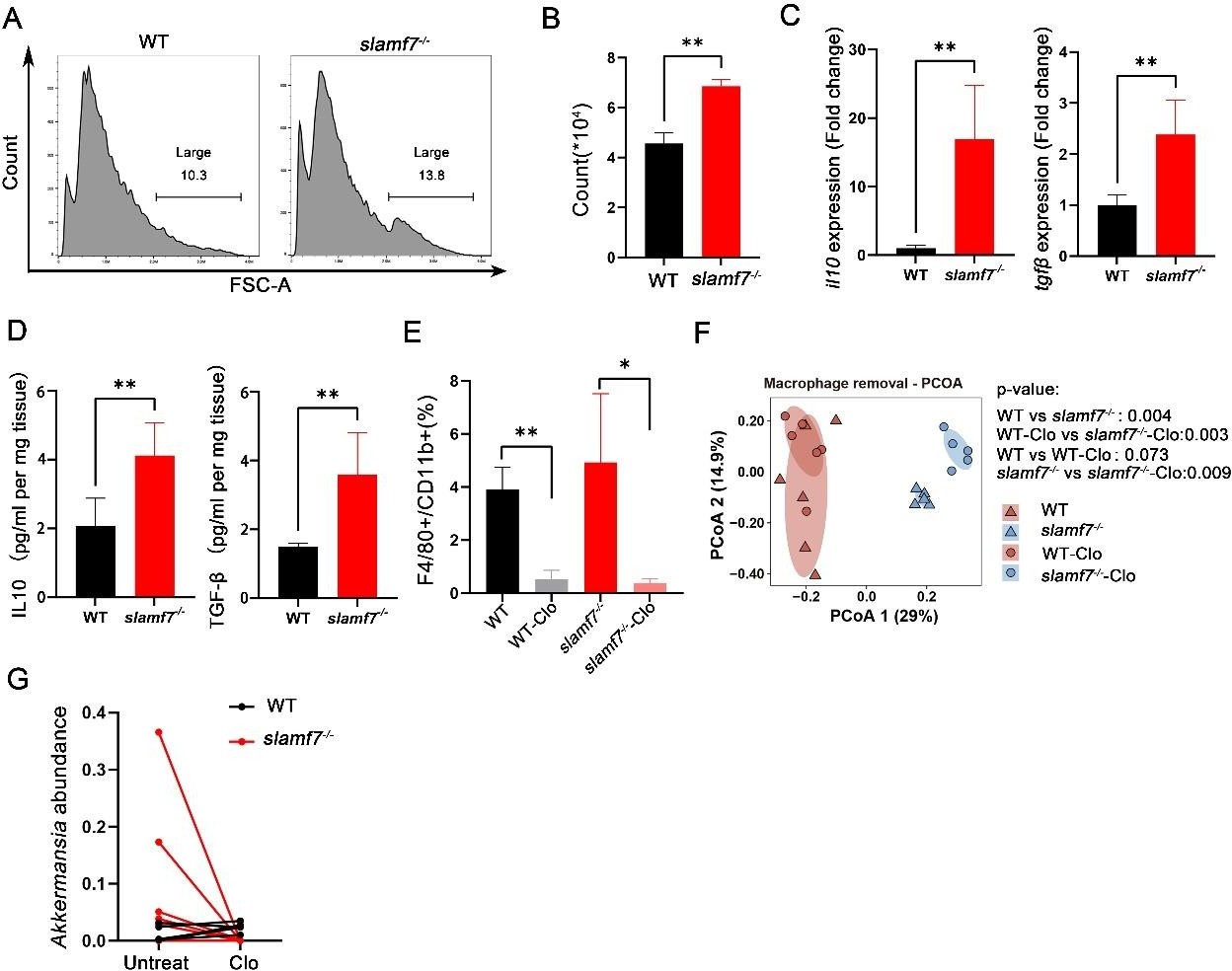


**Figure S9. Blocking IL-10 and TGF-β in the *slamf7^-/-^* mice intestine aggravated colitis.**

(A) 3D coculture of ISCs with intestinal macrophages from WT and *slamf7^-/-^* mice (n=3/group). *Slamf7^-/-^* intestinal macrophage medium from the *slamf7^-/-^*-Ab group was treated with IL-10 and TGF-β antibodies. The results following coculture for 3 days (upper) are shown; scale bar, 250 μm. The results following coculture for 5 days (below) are shown; scale bar, 50 μm.

(B-C) Enteroids were microscopically measured and quantified on day 7.

(D) A monolayer of Caco2 cells was cocultured with intestinal macrophages from WT or *slamf7^-/-^* mice. In the *slamf7^-/-^*-Ab group, intestinal macrophages were isolated from *slamf7^-/-^* mice and cultured with anti-IL-10 and anti-TGF-β antibodies; scale bar, 50 μm. The fluorescence intensity is quantified in (E).

(F) The *in vivo* RNA transfection murine model strategy is shown.

(G) IL-10 and TGF-β mRNA expression in the mouse intestine (n=5/group). Daily weight loss (H), DAI score (I), and colon length (J-K) were measured, and AB-PAS staining of colon tissues (L-M) was performed.

The data are presented as the means ± SEMs. ns, not significant, **p* < 0.05; ***p* < 0.01; ****p* < 0.001; *****p* < 0.001.


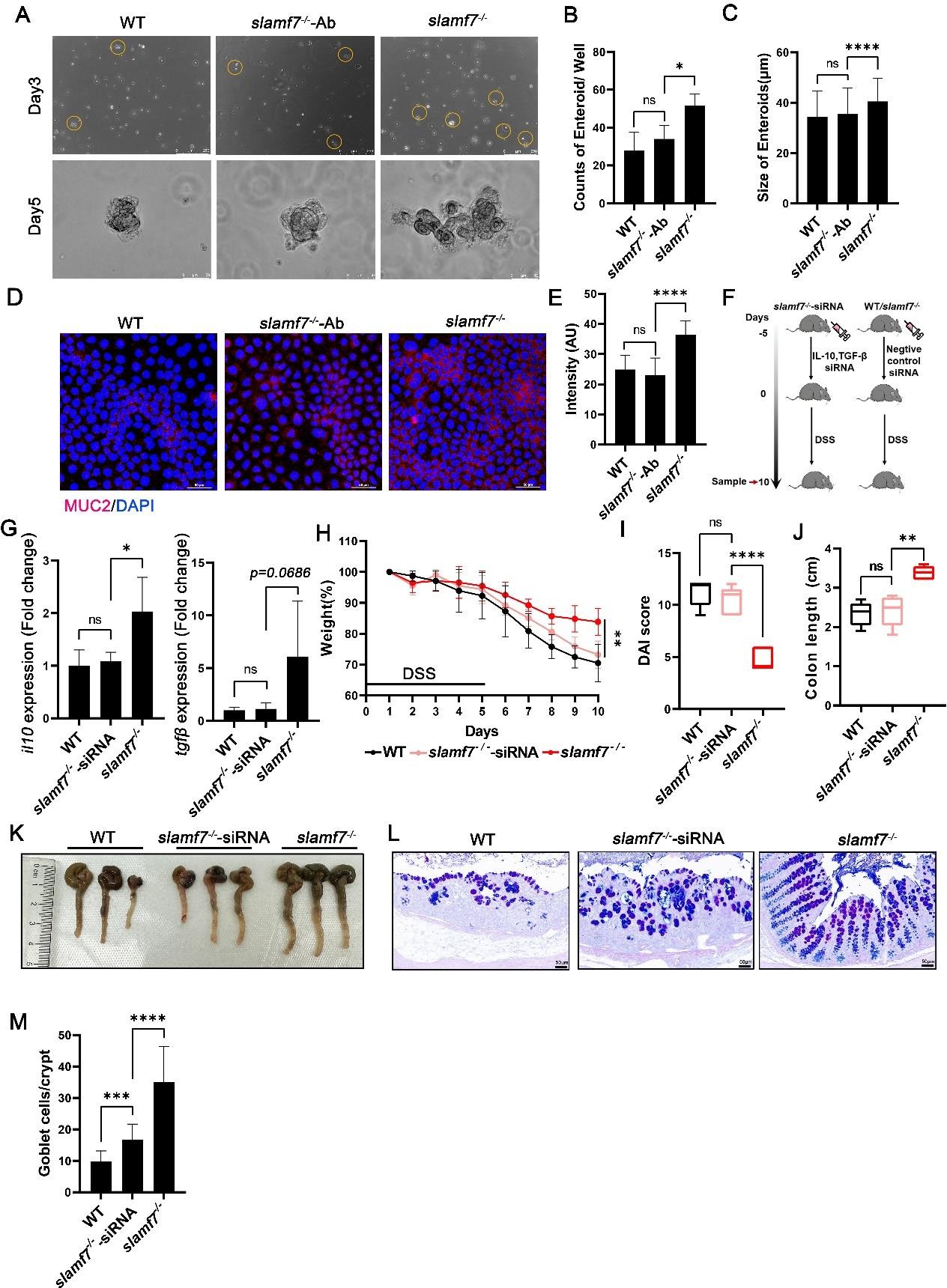


**Figure S10.** **SLAMF7 disrupted fibrin receptors in macrophage.**

(A) WT and *slamf7^-/-^* BMDMs were stimulated with fibrin (n=3/group), followed by qPCR to determine the expression of macrophage markers.

(B) qPCR was used to quantify MAC1 expression after SLAMF7 silencing in RAW264.7 cells (n=5/group).

(C) qPCR was used to quantify SLAMF7 and C1q expression after SLAMF7 and C1q silencing in RAW264.7 cells.

(D) qPCR was used to quantify SHP2 expression in WT and *slamf7^-/-^* BMDMs (n=3/group).

(E) RAW264.7 cells were transfected with HA-tagged SLAMF7. IP was used to detect the interaction of SLAMF7 and SHP2 with anti-HA and anti-SHP2 antibodies.

(F) RAW264.7 cells were transfected with siSLAMF7 and SHP2 plasmids and then stimulated with fibrin. Flow cytometry was used to analyze the percentage of CD86+ or CD206+ macrophages (n=3/group).

The data are presented as the means ± SEMs. ns, not significant, ***p* < 0.01; ****p* < 0.001; *****p* < 0.001.


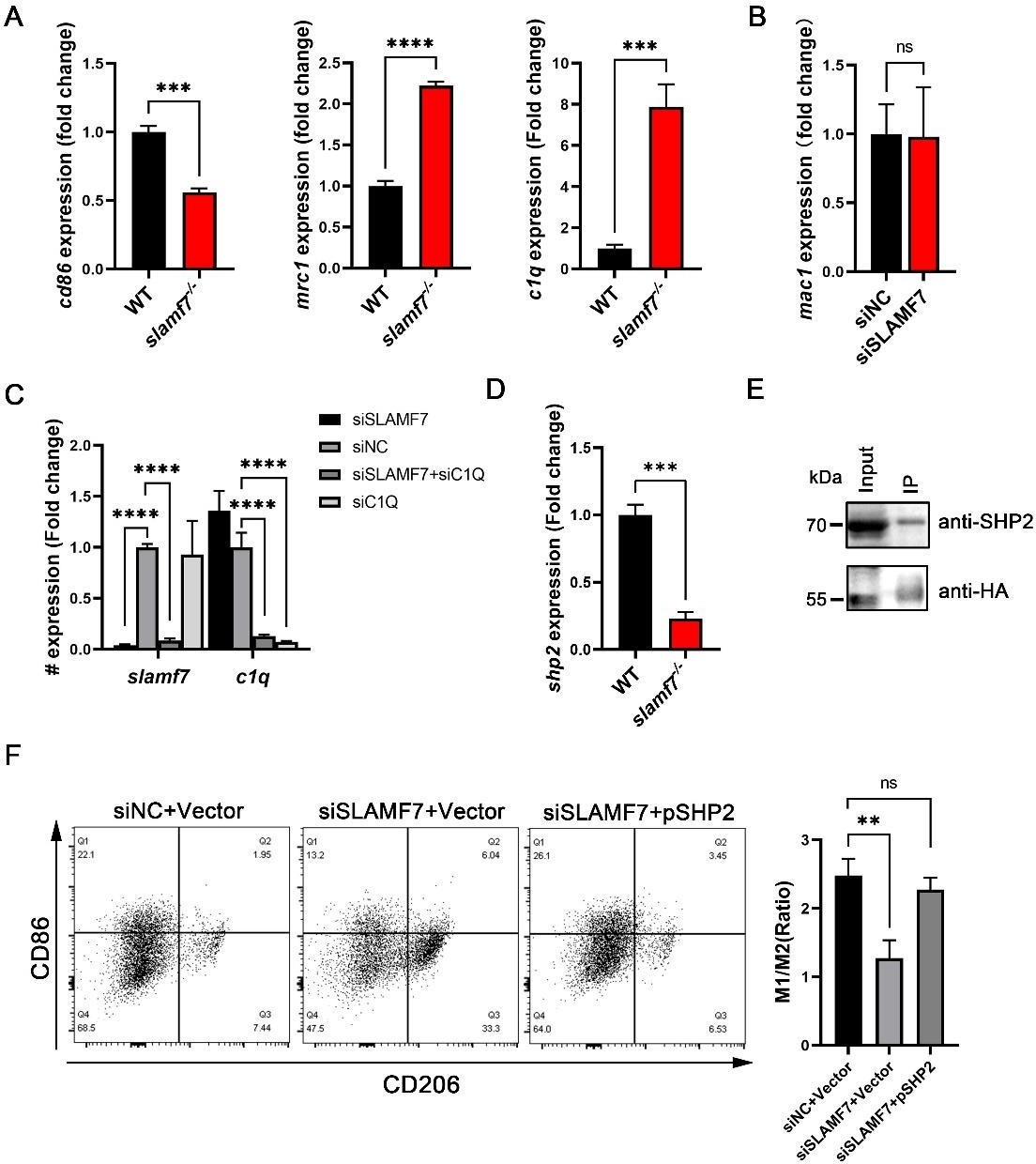


**Figure S11. SLAMF7 activation promoted colitis.**

Intestinal administration of recombinant SLAMF7 to WT and *slamf7*^-/-^ mice. (A) Weight loss, (B) DAI score.


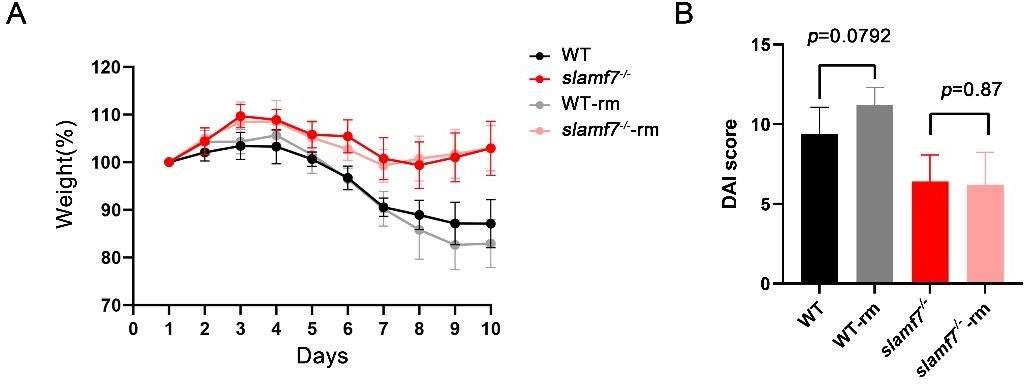


**Figure S12. The gating strategies of flow cytometry.**

Flow cytometry analysis: Gating strategies for CD86+ and CD206+ cells (A), MUC2+ cells (B), and C1q+ cells (C). Macrophage sorting: The gating strategy for F4/80 macrophages (D).


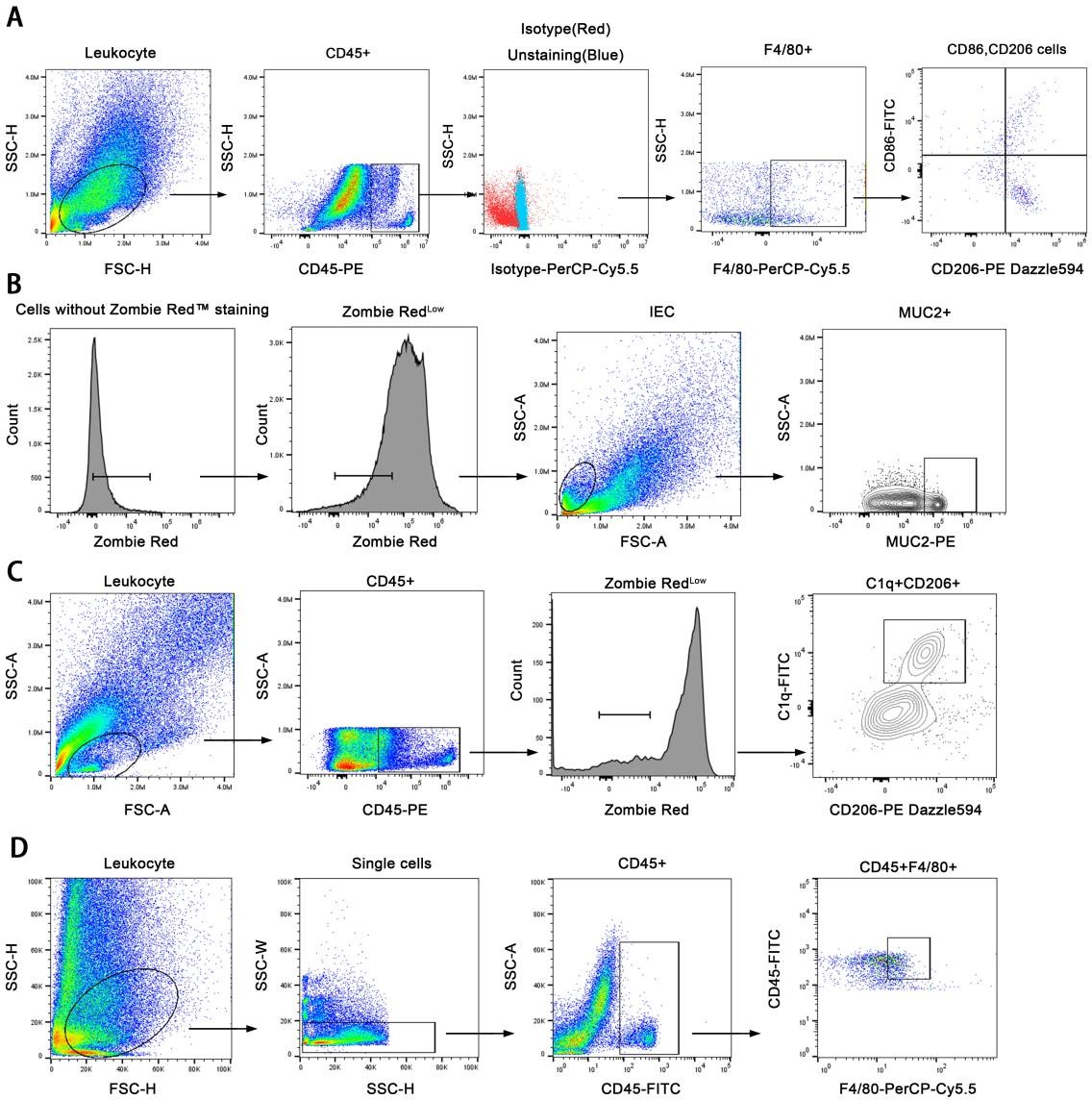


### Supplemental Materials and Methods

**Experimental colitis**

Experimental colitis was initiated by treating the mice with 2% dextran sulfate sodium (DSS) (36,000–50,000 M.Wt, MP Biomedicals) in autoclaved drinking water for 5 days. The DSS-containing water was then replaced with normal autoclaved water for 5 days. Body weight was monitored daily, and the disease-associated index (DAI) was determined on the basis of the following parameters: weight loss (0 points = 0% weight loss from baseline; 1 point = 1–5% weight loss; 2 points = 5–10% weight loss; 3 points = 10–20% weight loss; and 4 points = more than 20% weight loss), rectal bleeding (0 points = negative; 2 points = positive hemoccult test; and 4 points = gross bleeding), and stool consistency (0 points = normal; 2 points = semiformed stool; and 4 points = liquid that adheres to the anus). The sum of these three parameters resulted in a total DAI score ranging from 0 (healthy) to 12 (maximal level of colitis)^1^. On the tenth day, all the mice were euthanized, and the colon length was measured. The feces were collected on days 0 and 10.

**Microbiome removal from model mice**

Broad-spectrum antibiotics (ABXs) were prepared as previously described^69^. Briefly, ampicillin (0.5 g/l), vancomycin (0.25 g/l), metronidazole (0.5 g/l) and neomycin (0.5 g/l) were added to 1 packet of artificial sweetener in 250 ml of sterile drinking water. Magnetic stirring and gentle heating (37°C) were used to increase the antibiotic solubility. Antibiotic solutions were freshly prepared before each experiment. The pH was maintained within the physiological pH range (7.0~7.4). The mice were fed ABX for 1 week. Experimental colitis was initiated by treating the mice with 2% DSS. On the tenth day, all the mice were euthanized, and the colon length was measured. The feces were collected on day 0 and before ABX feeding.

**Littermate mouse cohousing model**

Littermate mice originating from the same breeders were divided into groups of individuals who were housed separately (WT-Siho and *slamf7^-/-^*-Siho) or cohoused (WT-Coho and *slamf7^-/-^*-Coho) with age- and sex-matched mice for 6 weeks. Cohoused mice were compared with their separately housed littermates as controls. Then, 2% DSS was used to induce experimental colitis. On the tenth day, all the mice were euthanized, and the colon length was measured. The feces were collected on day 0 and before cohousing.

**Fecal suspension preparation:**

A fecal suspension was prepared as previously described^70^. Feces were harvested directly from the anus of *slamf7^-/-^* and WT mice (donors) in a 1.5-ml Eppendorf tube in the morning. Fifty milligrams of feces (2-3 fecal pellets) was collected from each mouse. Fifty milligrams of donor feces was placed in sterile PBS in a 10-ml tube and incubated for 10 min. The feces were homogenized with a vortex mixer for 5 min–10 min. The samples were centrifuged at 800×g for 1 min. The supernatant was harvested for subsequent experiments. The fecal suspensions were collected fresh for every experiment.

**Fecal transplantation model**

*slamf7**^-/-^*→WT (WT) or WT→*slamf7^-/-^* (*slamf7^-/-^*) mice were fed ABX for one week. *slamf7^-/-^*→WT mice were orally gavaged for 3 weeks with 100 µl of a PBS suspension of feces (0.05 g/ml) obtained from *slamf7^-/-^* mouse donors, and WT→*slamf7^-/-^* mice were orally gavaged for 3 weeks with 100 µl of a PBS suspension of feces (0.05 g/ml) derived from WT mouse donors. WT and *slamf7^-/-^* mouse donors were used as controls. Then, 2% DSS was used to induce experimental colitis. On the tenth day, all the mice were euthanized, and the colon length was measured. The feces were collected on day 0 and before fecal transplantation.

**Microbiota rebuilding model**

WT or *slamf7^-/-^* mice were fed ABX for one week and conventionally raised for 4 weeks. The feces were collected on days 7 and 21.

**Intestinal administration method**

Intestinal administration was performed as previously described^71,72^. The mice were fasted for 12 hours prior to injection, and gentle abdominal massage was performed to stimulate bowel movement and ensure complete intestinal clearance. The administered solution was prepared by the following methods. The mouse was placed in a mouse restraint device. The tip of a sterile intestinal catheter was carefully inserted tip into the mouse anus and gently advanced approximately 2–3 cm into the colon. The solution (50 µl per mouse) was slowly delivered into the colon to ensure precise dosing.

**Colonic macrophage removal model**

WT (WT-clo) and *slamf7^-/-^* (*slamf7^-/-^*-clo) mice were intraperitoneally injected with 120 µl/per 20 g of neutral clodronate liposomes (FormuMax). WT and *slamf7^-/-^* mice were intraperitoneally injected with 120 µl/per 20 g of control liposomes as a control and then conventionally raised for 4 days. DSS (2%) was used to induce experimental colitis.

***In vivo* transfection experiment**

We utilized a commercial *in vivo* transfection reagent (Engreen Biosystem Co., Ltd., Cat. No. 18668-11-1) for long-term gene silencing. The target gene was silenced *in vivo* according to the manufacturer’s instructions, with a final concentration of 1 µg/µl siRNA in a total volume of 30 µl. To maintain sustained silencing, intestinal administration was performed every two days for one week. *Slamf7^-/-^* mice were randomly assigned to either the *slamf7^-/-^*-siRNA group or the *slamf7^-/-^* control group (n = 5/group). Mice in the *slamf7^-/-^*-siRNA group received IL-10 and TGF-β siRNA via intestinal administration, whereas mice in the WT and *slamf7^-/-^* control groups were treated with control siRNA. Experimental colitis was then induced via 2% DSS.

**SLAMF7 activation experiment**

The intestinal administration of recombinant SLAMF7 was performed as previously described^16,17,73^. A working solution was prepared by dissolving 40 µg of recombinant SLAMF7 (R&D Systems) in 1 ml of sterile 2% DSS solution. Each mouse received 2 µg of recombinant SLAMF7 (50 µl of the working solution) per 20 g of body weight via intestinal administration. The mice were divided into the following groups: the WT-rm and *slamf7^-/-^*-rm groups, in which recombinant SLAMF7 was administered intestinally; and the WT and *slamf7^-/-^* control groups, in which 2% DSS alone was administered intestinally. Recombinant SLAMF7 was administered intestinally once a day for five days.

**H&E and AB/PAS staining analysis**

For evaluation of mucus thickness, colon sections containing stool pellets were collected and fixed in 4% PFA or Carnoy's Fixative for at least 24 hours prior to H&E (hematoxylin and eosin) and AB/PAS (alcian blue/periodic acid-Schiff) staining, and images were collected by a Pannoramic MIDI. Histology scores were determined by a pathologist. The scoring system evaluates inflammation, epithelial defects, the area of inflammation, the area of epithelial defects, crypt atrophy, and dysplasia-neoplasia. Each parameter was graded on a scale of 0 to 4, reflecting the severity and extent of the changes, as previously described^1^. For AB/PAS staining, we measured the mucus layer thickness and determined the number of goblet cells per crypt using CaseViewer. The thickness of the mucus layer was measured at ten randomly selected locations where the mucosa appeared continuous and intact, and the average value was calculated^74^.

**Histological analysis**

Slides of mouse colon tissue were deparaffinized with xylene, and antigen retrieval was conducted for 20 min in a 95°C water bath in 10 mM sodium citrate (pH 6.0), followed by 15 min of cooling to room temperature. The slides were washed with PBS, blocked in 5% BSA or 10%FBS for 60 min to 120min, and stained with the primary antibodies rabbit anti-claudin4 (1:200, Servicebio), rabbit anti-ZO1 (1:200, Servicebio), rabbit anti-MUC2 (1:500, Servicebio), and rabbit anti-Ki67 (1:1000, Servicebio) and with secondary antibodies conjugated to CY3 (1:300, Servicebio) and Alexa Fluor 488 (1:400, Servicebio) diluted in PBS buffer containing 2% BSA at 4°C. Finally, the slides were incubated with DAPI solution at room temperature for 10 min and kept in the dark. Mouse intestinal stem cells were plated onto coverslips in 24-well plates and incubated with 500 μl of 4% paraformaldehyde for 15 min at room temperature. After the coverslips were washed twice with PBS, the samples were permeabilized for 15 min in PBS buffer containing 1% Triton X-100 and 5% BSA, washed, and then blocked with 5% BSA for an additional hour at room temperature. The samples were then incubated with primary rabbit anti-MUC2 antibody (1:200, Affinity) diluted in PBS buffer containing 2% BSA at 4°C overnight. After being washed three times, the samples were treated with secondary antibody conjugated to Cy3 (1:500, Proteintech) diluted in PBS buffer containing 2% BSA for 1 hour at room temperature in the dark. After three washes with PBS, the coverslips were mounted with DAPI solution at room temperature for 10 min. The sections were subsequently imaged using Nikon Eclipse C1, Nikon DS-U3, Nikon C2.

**Single-cell sample preparation and sequencing**

1.1 Cell preparation

The 10x Genomics^®^ Cell Preparation Guide describes best practices and general protocols for washing, counting and concentrating cells from both abundant and limited cell suspensions (greater than or less than 100,000 total cells, respectively) in preparation for 10x Genomics single-cell protocols <https://www.10xgenomics.com/support>**.**

1.2 Single-cell RNA sequencing (scRNA-seq)

The cell suspension was loaded into chromium microfluidic chips with 3’ (v2 or v3, depending on the project) chemistry and barcoded with a 10× chromium controller (10X Genomics). RNA from the barcoded cells was subsequently reverse-transcribed, and sequencing libraries were constructed with reagents from a Chromium Single Cell 3’ (v2 or v3, depending on project) reagent kit (10X Genomics) according to the manufacturer’s instructions. Sequencing was performed with an Illumina instrument (NovaSeq) according to the manufacturer’s instructions (Illumina), with support from Chi Biotech Co., Ltd.

**Single-cell sequencing data analysis**

We used fastp to perform quality control of the raw reads. The raw reads were demultiplexed and mapped to the reference genome by the 10× Genomics Cell Ranger pipeline using default parameters. All downstream single-cell analyses were performed using Cell Ranger and Seurat^75^ unless otherwise indicated. In brief, for each gene and each cell barcode (filtered by Cell Ranger), unique molecule identifiers were counted to construct digital expression matrices. Secondary filtration by Seurat: A gene with expression in more than 3 cells was considered expressed, and each cell was required to have at least 200 expressed genes. Some of the foreign cells were filtered out. Seurat: The Seurat package was used to normalize the data for dimensionality reduction, clustering, and differential expression determination. For clustering, highly variable genes were selected, and the principal components based on those genes were used to construct a graph, which was segmented with a resolution of 0.6. Enrichment analysis of marker genes: Gene Ontology (GO) enrichment analysis of marker genes was implemented by the clusterProfiler R package, in which gene length bias was corrected. GO terms with corrected P values less than 0.05 were considered significantly enriched with marker genes. We used the clusterProfiler R package to test the statistical enrichment of marker genes in KEGG pathways.

For determining differential cell type abundances, the Wilcoxon rank-sum test and Student’s t test were used to compare two groups on the basis of the results of the Shapiro‒Wilk test. All hypothetical tests were two-sided, and a p value < 0.05 indicated significance. For the cell type compositional analysis, we utilized t-distributed stochastic neighbor embedding (t-SNE) to visualize high-dimensional data in two dimensions, allowing for the identification of distinct cell clusters. The t-SNE plots were generated on the basis of the expression profiles of various cell types, with each dot representing a single cell and colors indicating different cell types. For determining differential cell type abundance, the Wilcoxon rank-sum test and Student’s t test were used to compare two groups on the basis of the results of the Shapiro‒Wilk test. All hypothetical tests were two-sided, and a *p* value < 0.05 indicated significance. Additionally, we assessed the expression levels of specific genes (C1qa, C1qb, and MRC1) across different cell types using violin plots and bar charts. The bar charts represent the percentage of nonzero expression for each gene in the C1qaC1qb macrophage population. Statistical comparisons between two groups of samples were made using appropriate tests, such as the Wilcoxon rank-sum test or t test, to evaluate the significance of gene expression differences.

**Fecal** **16S amplicon sequencing and analysis**

DNA was extracted from the corresponding samples using a DNA extraction kit. The concentration and purity were measured using a NanoDrop One system (Thermo Fisher Scientific). The 16S rRNA genes were amplified using specific primers (806R/515F) with 12 bp barcodes. The primers used were synthesized by Invitrogen (Invitrogen).

The sequencing libraries were generated using the NEBNext^®^ Ultra™ II DNA Library Prep Kit for Illumina^®^ (New England Biolabs) according to the manufacturer's recommendations, and index codes were added. Library quality was assessed on a Qubit@ 2.0 fluorometer (Thermo Fisher Scientific, MA, USA). Finally, the library was sequenced on an Illumina NovaSeq 6000 platform, and 250-bp paired-end reads were generated (Guangdong Magigene Biotechnology Co.).

Paired-end reads were processed using the QIIME2 pipeline v2019.7 ^76^. The raw sequences were subjected to quality filtering, denoising, chimera removal, and demultiplexing with DADA2 under the parameters ‘-p-trunc-len-f 248’ and ‘-p-trunc-len-r 236’^77^. Unique amplicon sequence variants (ASVs) were taxonomically classified against the SILVA v.132 rRNA database^78^. Finally, an OTU table was generated along with the corresponding taxonomic classifications.

For downstream statistical analyses, R (v4.2.1) and specialized packages were employed. Alpha diversity was estimated using the Shannon index via the ‘phyloseq’ package. Beta diversity was quantified using Bray–Curtis dissimilarity and statistically evaluated through PERMANOVA (permutational multivariate analysis of variance) using the ‘adonis’ function in the ‘vegan’ package. Additionally, differential taxon identification was performed using linear discriminant analysis effect size (LEfSe) v1.0 on a Linux-based platform^79^, with the significance threshold set at *p* < 0.05.

Visualization workflows included microbial community heatmaps generation with the ‘pheatmap’ package, differential abundance volcano plots creation via the ‘EnhancedVolcano’ package, and general graphics production using ‘ggplot2’.

**Untargeted metabolomic analysis**

1.1 Stool sample preparation and extraction

Stool samples from WT and *slamf7^-/-^* mice were collected at steady state and stored at -80°C. After thawing on ice, 400 μL of solution (methanol:water =7:3, V/V) containing an internal standard was added to 20 mg of sample and vortexed for 3 min. The sample was sonicated in an ice bath for 10 min, vortexed for 1 min, and then placed at -20°C for 30 min. The sample was then centrifuged at 12,000 rpm for 10 min (4°C). The sediment was removed, and the supernatant was subsequently centrifuged at 12,000 rpm for 3 min (4°C). Two hundred-microliter aliquots of the supernatant were transferred for LC‒MS analysis.

1.2 HPLC conditions (T3)

All samples were analyzed via an LC‒MS system according to the manufacturer’s instructions. The analytical conditions were as follows: Waters ACQUITY UPLC HSS T3 C18 column (1.8 µm, 2.1 mm*100 mm); 40°C column temperature; 0.4 mL/min flow rate; 2 μL injection volume; and 0.1% formic acid:acetonitrile (0.1% formic acid) mixture. The column was eluted with 5% mobile phase B (0.1% formic acid in acetonitrile) at 0 min, followed by a linear gradient to 90% mobile phase B (0.1% formic acid in acetonitrile) over 11 min, held for 1 min, returned to 5% mobile phase B within 0.1 min, and held for 1.9 min.

**Intestinal epithelial cell and immune cell isolation**

The distal colon was collected from euthanized mice and flushed with ice-cold PBS. The colon samples were cut into 0.5-cm pieces and incubated at 37°C for 30 min in Ca/Mg-free HBSS (Sangon Biotech) containing 5 mM EDTA, 15 mM HEPES (Biosharp), and 1% FBS (HyClone). After vigorous shaking, the wash mixture containing intestinal epithelial cells and immune cells was collected. This step was performed three times for the colon pieces, and the cells were collected at the end of each wash. For lamina propria immune cell isolation, colon pieces after EDTA treatment, as described above, were digested in 10 mL of HBSS containing 1% FBS, 1 mg/mL collagenase VIII (Sigma) and 100 U/mL DNase I (Sigma) for approximately 40 min at 37°C with shaking. The digested tissue was filtered through a 40-mm strainer. The cells were resuspended in PBS containing 2% FBS for flow cytometry.

**Flow cytometry analysis**

Mouse intestinal epithelial single-cell suspensions were incubated at 4°C with rabbit anti-MUC2 (1:100, Abcam) for 1 h and washed twice with flow buffer. Then, Cy3-conjugated secondary antibody (1:200, Proteintech) and Zombie red (BioLegend) were added, and the samples were incubated at room temperature for 30 min and washed twice with flow buffer. Monoclonal antibodies conjugated with fluorescein were purchased from BioLegend (1:100, anti-mouse CD4, CD8, CD3, CD19, CD86, CD45, CD206, CD11b, CD11c, Ly-6G/Ly-6C, NK1.1, F4/80, and C1qa; details are shown in Supplemental Table 1). For the lamina propria and cell lines, immune cells were incubated at 4°C with monoclonal antibodies (mixed with True-Stain Monocyte Blocker™, BioLegend) for 1 hour and washed twice with flow buffer. For isotype control preparation, the cell suspensions above were divided into an additional aliquot for isotype control staining. Rat IgG2a and κ isotype control antibodies (1:100, BioLegend) were added to the isotype control samples at the same concentration as the experimental antibodies, and the isotype control samples were processed following the same protocol as used for the experimental antibodies. For flow cytometry analysis, at least 100,000 live samples were analyzed using Cytek AURORA (Aurora) and FlowJo software (v10.6.2). FSC and SSC were used to distinguish cell populations. Zombie red was used to exclude dead cells. The isotype control sample was used to set the threshold of the fluorescence channels. CD45+ cells were gated to identify leukocytes. M1 macrophages were defined as F4/80+CD86+ cells. M2 macrophages were defined as F4/80+CD206+ cells. MUC2+ cells were gated to identify goblet cells^33,34^. C1q+CD206+ cells were gated to identify C1q+M2-like macrophages (Figure S12A-C). The antibodies used in our study are listed in Supplemental Table 1.

**Macrophage sorting**

WT and *slamf7^-/-^* mouse colon single-cell suspensions were collected following the above protocols. Sorting was performed on MoFlo Astrios EQs system (Beckman Coulter). The single-cell suspensions were incubated with anti-mouse CD45-FITC and anti-mouse F4/80-PerCP/Cy5.5 monoclonal antibodies. The gating strategy was as follows: draw a gate on forward scatter (FSC) vs. side scatter (SSC) to exclude debris and dead cells; use SSC-H vs. SSC-W to exclude doublets or cell aggregates; draw a gate on CD45+ cells; and harvest the F4/80+ population (Figure S12D).

**3D colonoid, 2D primary epithelial, and mouse macrophage cocultures**

The mouse intestinal stem cell line and primary epithelial cell culture system were purchased from iCell Bioscience (MIC-iCell-d032 and PriMed-iCell-001). Macrophages were isolated from the colon of WT and *slamf7^-/-^* mice (age- and sex-matched) according to the methods previously described in this manuscript. The intestinal stem cells used in each experiment were maintained at the same passage (within five passages). For 3D colonoid and macrophage coculture^33,61,80^, the epithelial cell culture medium was prepared by mixing 3× additives (3×2% FBS, 3×penicillin/streptomycin, 3×50 ng/ml murine EGF, 3×100 ng/ml murine Wnt3a, and 3×100 ng/ml Noggin). A total of 5×10^4^ macrophages and 1×10^4^ mouse intestinal stem cells were resuspended together in a VitroGel hydrogel matrix (well VHM01), mixed with an epithelial cell culture system at a 2:1 v/v mixing ratio, and transferred into 48-well plates (300 µl/well). In the control group, intestinal stem cells were cultured alone in the absence of macrophages. The samples were left for 10–15 min at room temperature to allow for soft gel formation, and the hydrogels were carefully covered with 150 µl of epithelial cell medium. For cell recovery from the hydrogel, the VitroGel Cell Recovery Solution (The Well, MS03-100) was warmed to 37°C, and 1 ml of solution was added to each well. After the hydrogel was dissolved into small pieces, 5 ml of warm VitroGel Cell Recovery Solution was added to a 15-ml conical tube, after which the hydrogel was transferred to the tube. The mixture was gently pipetted 3–5 times, and the tube was returned to the water bath for 2–3 min. The samples were subsequently centrifuged at 100 × g for 3–5 min at room temperature to collect the cell pellet. For 2D monolayer cultures, 1×10^3^ mouse intestinal stem cells suspended in DMEM (5% FBS, penicillin/streptomycin) were plated in 48-well plates/well and placed in an incubator. Two days later, the medium of the mouse intestinal stem cells was removed. Macrophages (5×10^4^) were suspended in an epithelial cell culture system and added to a monolayer of mouse intestinal stem cells. The plate was placed in an incubator for 1 week. The samples were imaged using an Olympus BX63 microscope.

***In vivo* anti-IL-10 and anti-TGF-β treatment**

To block the secretion of IL-10 and TGF-β from macrophages, the culture supernatants from *slamf7^-/-^* intestinal macrophages were incubated with anti-IL-10 (BioLegend) and anti-TGF-β (BioLegend) antibodies. The concentration of the antibody was 1 µg/ml.

**ELISA sample preparation**

The tissue was placed in a 1.5-ml tube and weighed. Next, 0.5 mL of cold PBS was added to each tube. The tissues were ground for 6 min at 4°C to facilitate cytokine release and then centrifuged at 15,000–17,000 × g for 10 min at 4°C to pellet the insoluble contents. The supernatant (soluble cell extract) was transferred to clean, chilled tubes on ice. The supernatant was diluted 10 times for detection. IL-10 and TGF-β in colon tissues were detected following the ELISA kit protocol (Neobio Science). The main steps of the ELISA protocol were as follows. Sample and standard addition: The supernatant and standard solutions (IL-10: 250, 125, 62.5, 31.25, 15.6, 7.8, 3.9, and 0 pg/ml; TGF-β: 2000, 1000, 500, 250, 125, 62.5, 31.25, and 0 pg/ml) were added to the designated wells. The plate was incubated at 37°C for 90 minutes. Biotinylated secondary antibody incubation: The plate was incubated with the biotinylated secondary antibody at 37°C for 60 minutes. Enzyme conjugate and substrate addition: The enzyme conjugate reagent was added to the wells, followed by the substrate mixture, to initiate the enzymatic reaction. Reaction termination and measurement: Stop solution was added to terminate the reaction, and the optical density (OD) was measured at 450 nm within 3 minutes.

**Quantitative real-time PCR analysis**

Total RNA was isolated from mouse colon tissue with RNA Isolater Total RNA Extraction Reagent (Vazyme). cDNA was synthesized using HiScript III RT SuperMix for qPCR (Vazyme). The mRNA levels of the selected targets were quantified via qPCR using ChamQ Universal SYBR qPCR Master Mix (Vazyme) and specific primers and were normalized to that of β-actin. The primer list is shown in Supplemental Table 1**.** The experimental validation data of the primers were obtained from PrimerBank (<https://pga.mgh.harvard.edu/primerbank/>). The procedures were performed according to the manufacturer’s instructions. The qRT‒PCR parameters used were as follows. Stage 1: initial denaturation at 95°C for 30 s; stage 2 (40 cycles): denaturation at 95°C for 10 s, annealing at 60°C for 30 s; and stage 3: melting curve at 65°C to 95°C, increasing incrementally (0.5°C every 5 s). The software was allowed to automatically set the threshold.

**Preparation of colonic epithelial cell-derived DAMPs**

Caco2 cells were harvested by trypsinization, and 1 × 10^6^ cells were resuspended in 500 µl of PBS, followed by five freeze‒thaw cycles. The cells were spun at 1000 × g at 4°C to collect the supernatant and were then stored at −80°C for further use^40^. RAW264.7 cells were cultured in a 6-well plate until the cell density reached 60~70%. EDD was used at 100 μl/well to stimulate the RAW264.7 cells for 12 h.

**Cell culture and stimulation**

A total of 1 × 10^6^ RAW264.7 cells per well were cultured in 6-well plates in DMEM (Thermo Fisher Scientific) supplemented with 10% FBS and penicillin/streptomycin. The cells were allowed to rest for 24 hours. For DAMP stimulation, after 12 hours of siRNA transfection, RAW264.7 cells were stimulated with 2.5 µg/ml DAMPs (MedChemExpress) for 12 hours. For the inhibitor experiment, after 6 hours of siRNA transfection, RAW264.7 cells were treated with 2.4 nM AS1810722 (TargetMol) for 12 hours. The expression of macrophage markers was analyzed.

**BMDM isolation and stimulation**

WT and *slamf7^-/-^* mice were sacrificed and thoroughly soaked in 70% ethanol. Using sterile scissors and forceps, a leg was carefully removed by cutting at the hip joint, ensuring that the femur remained intact. Muscle tissue was removed from the bones, and the femur was carefully separated from the tibia, keeping both bones intact. The bones were dip-cleaned in 70% ethanol and placed in a Petri dish. After a 10-ml syringe was then filled with ice-cold PBS, the needle was inserted into the bone cavity, and the marrow was flushed with PBS into a 15-ml tube. The cells were subsequently centrifuged for 10 min at 500 × g and 4°C. The supernatant was removed, and the cells were resuspended in 10 ml of 1x RBC lysis buffer. A total of 1 × 10^6^ cells/well were plated in 2 ml of complete media containing 25 ng/mL M-CSF. After 3 days, the complete medium containing 2.5 µg/ml fibrin was replaced. Two days later, all BMDMs were harvested, and the expression of macrophage polarization markers was analyzed.

**Plasmid construction and transient transfection**

The cDNA sequences of murine SLAMF7 and SHP2 with a C-terminal tag were obtained by reverse transcription PCR, subcloned and inserted into the pcDNA3.1(+) vector. Small interfering RNAs (siSLAMF7 and siC1Q) were used to silence SLAMF7 and C1q in RAW264.7 cells. All siRNAs used were purchased from RiboBio. RAW264.7 cells were cultured in Opti-MEM (Thermo Fisher Scientific). For transient siRNA transfection, RAW264.7 cells were transfected with either a negative control siRNA or siRNA against target genes at 30 nM by Lipofectamine 3000 (Thermo Fisher Scientific). For transient plasmid transfection, RAW264.7 cells were transiently transfected with 2 µg of plasmid by Lipofectamine 3000.

**Western blotting and immunoprecipitation**

Protein from tissues or cell lines was solubilized in RIPA buffer (Biosharp). The supernatant was transferred to new tubes, and the proteins were quantified with a BCA protein assay kit (Beyotime). Equal amounts of lysate were mixed with SDS‒PAGE sample loading buffer (Biosharp) and boiled for 10 min at 95°C. SDS‒PAGE was performed using an 8% One-Step PAGE Gel Fast Preparation Kit (Vazyme). The membranes were visualized using a SuperPico ECL Chemiluminescence Kit (Vazyme) and a ChemiDoc Touch system (Bio-Rad). The following antibodies were used for immunoblotting: rabbit anti-MUC2 (1:1000, Affinity), mouse anti-actin (1:1000, ZSGB-BIO), HRP-labeled goat anti-rabbit (1:5000, ZSGB-BIO), and HRP-labeled rabbit anti-mouse (1:5000, Zennia) antibodies. For immunoprecipitation, 500 µl of RIPA buffer (Biosharp) per 50 mg of wet cell pellet was used to prepare lysates from RAW264.7 cells. A total of 10 µl of anti-HA (share-bio) purified antibody was combined with the cell lysate in a microcentrifuge tube. The amount of total protein per IP reaction was 1,000 µg. The mixture was incubated overnight at 4°C to form the immune complex. The magnetic beads were placed in a new collection tube, and 50 µl of 2X SDS‒PAGE sample buffer was added. The mixture was incubated at 100°C for 10 minutes. The proteins were detected using anti-HA (1:1000, Beyotime) and anti-SHP2 (1:1000, Affinity Biosciences) antibodies.

**Statistical analyses**

The results are presented as the mean ± SEM. The sample sizes for the mouse model experiments were chosen according to standard practices in the field^61,64^, and at least three biological replicates were conducted. The sample size for enteroid size determination was determined by GPower 3.1. The sample sizes (n) used in the experiments are indicated in the figure legend. Statistical analyses were performed using GraphPad Prism v.9.0. Significance between two groups was calculated using an unpaired two-tailed Student’s t test. Significance between multiple groups was determined using one-way analysis of variance (ANOVA) with Fisher’s LSD test. Statistical significance in survival studies was assessed using the log-rank test (Mantel‒Cox test). Differences were considered statistically significant when *p* values were <0.05. For all statistical comparisons, **p*<0.05, ***p*<0.01, ****p*<0.001 and *****p*< 0.0001.

**Data availability statement**

The 16S rRNA sequencing data in this study have been deposited in the National Center for Biotechnology Information (NCBI) database under project number PRJNA1020971. The 16S rRNA sequencing data of human IBD patients and healthy individuals were obtained from GenBank under project number PRJNA596333. The single-cell sequencing data in this study have been deposited in the NCBI database under project number PRJNA1020529.

References

69. Zhang W, Lyu M, Bessman NJ, et al. Gut-innervating nociceptors regulate the intestinal microbiota to promote tissue protection. *Cell* 2022; **185**(22): 4170-89.e20.

70. Bokoliya SC, Dorsett Y, Panier H, Zhou Y. Procedures for Fecal Microbiota Transplantation in Murine Microbiome Studies. *Front Cell Infect Microbiol* 2021; **11**: 711055.

71. McCoubrey LE, Favaron A, Awad A, Orlu M, Gaisford S, Basit AW. Colonic drug delivery: Formulating the next generation of colon-targeted therapeutics. *J Control Release* 2023; **353**: 1107-26.

72. Li J, Dejanovic D, Zangara MT, Chandra J, McDonald C, Rieder F. Mouse Models of Intestinal Fibrosis. Myofibroblasts; 2021: 385-403.

73. Yan F, Cao H, Cover TL, et al. Colon-specific delivery of a probiotic-derived soluble protein ameliorates intestinal inflammation in mice through an EGFR-dependent mechanism. *J Clin Invest* 2011; **121**(6): 2242-53.

74. Röhe I, Hüttner FJ, Plendl J, Drewes B, Zentek J. Comparison of different histological protocols for the preservation and quantification of the intestinal mucus layer in pigs. *European Journal of Histochemistry* 2018.

75. Macosko EZ, Basu A, Satija R, et al. Highly Parallel Genome-wide Expression Profiling of Individual Cells Using Nanoliter Droplets. *Cell* 2015; **161**(5): 1202-14.

76. Bolyen E, Rideout JR, Dillon MR, et al. Reproducible, interactive, scalable and extensible microbiome data science using QIIME 2. *Nat Biotechnol* 2019; **37**(8): 852-7.

77. Callahan BJ, McMurdie PJ, Rosen MJ, Han AW, Johnson AJ, Holmes SP. DADA2: High-resolution sample inference from Illumina amplicon data. *Nat Methods* 2016; **13**(7): 581-3.

78. Pruesse E, Quast C, Knittel K, et al. SILVA: a comprehensive online resource for quality checked and aligned ribosomal RNA sequence data compatible with ARB. *Nucleic Acids Res* 2007; **35**(21): 7188-96.

79. Segata N, Izard J, Waldron L, et al. Metagenomic biomarker discovery and explanation. *Genome Biol* 2011; **12**(6): R60.

80. Zhu P, Lu T, Wu J, et al. Gut microbiota drives macrophage-dependent self-renewal of intestinal stem cells via niche enteric serotonergic neurons. *Cell Res* 2022; **32**(6): 555-69.
